# Gene Editing in Rat Embryonic Stem Cells to Produce *In Vitro* Models and *In Vivo* Reporters

**DOI:** 10.1016/j.stemcr.2017.09.005

**Published:** 2017-10-10

**Authors:** Yaoyao Chen, Sonia Spitzer, Sylvia Agathou, Ragnhildur Thora Karadottir, Austin Smith

**Affiliations:** 1 Wellcome Trust-Medical Research Council Cambridge Stem Cell Institute, University of Cambridge, Tennis Court Road, Cambridge CB2 1QR, UK; 2 Department of Veterinary Medicine, University of Cambridge, Madingley Road, Cambridge CB3 0ES, UK; 3 Department of Biochemistry, University of Cambridge, Tennis Court Road, Cambridge CB2 1GA, UK

**Keywords:** gene targeting, CRISPR/Cas9, oligodendrocyte, neural crest, rat, transgenesis, homologous recombination, pluripotency, SOX10, embryonic stem cells

## Abstract

Rat embryonic stem cells (ESCs) offer the potential for sophisticated genome engineering in this valuable biomedical model species. However, germline transmission has been rare following conventional homologous recombination and clonal selection. Here, we used the CRISPR/Cas9 system to target genomic mutations and insertions. We first evaluated utility for directed mutagenesis and recovered clones with biallelic deletions in *Lef1*. Mutant cells exhibited reduced sensitivity to glycogen synthase kinase 3 inhibition during self-renewal. We then generated a non-disruptive knockin of *dsRed* at the *Sox10* locus. Two clones produced germline chimeras. Comparative expression of dsRed and SOX10 validated the fidelity of the reporter. To illustrate utility, live imaging of dsRed in neonatal brain slices was employed to visualize oligodendrocyte lineage cells for patch-clamp recording. Overall, these results show that CRISPR/Cas9 gene editing technology in germline-competent rat ESCs is enabling for *in vitro* studies and for generating genetically modified rats.

## Highlights

• Gene mutation and homologous recombination in rat ESCs using CRISPR/Cas9
• *Lef1* mutants exhibit predicted loss of hypersensitivity to GSK3 inhibition
• *Sox10* knockin rat provides a vital reporter of neural crest and oligodendroglia
• *Sox10::dsRed* facilitates patch-clamp recording from oligodendroglial lineage cells

## Teaser

In this article, Smith and colleagues demonstrate that diploid clones of rat embryonic stem cells can reliably be recovered after CRISPR/Cas9-facilitated gene disruption or knockin and subsequently used for *in vitro* studies or to generate genetically engineered rats.

## Introduction

The rat *Rattus* is a valuable and widely used model organism for studying cognition and behavior, physiology, toxicology, and various pathologies, such as metabolic and neurodegenerative diseases (Iannaccone and Jacob, 2009). Although the rat was the first mammalian species to be domesticated for biomedical research (Jacob et al., 2010), it has been outpaced in recent years by the mouse, in part because of limitations in directed manipulation of the rat genome. In mice, genome engineering is mostly performed via embryonic stem cells (ESCs), and the ease of carrying out such work has been key to their widespread use as an animal model (Capecchi, 2005). Following the definition of culture requirements for mouse ESCs (Ying et al., 2008), rat ESCs have been derived from different rat strains using similar conditions (Buehr et al., 2008, Hirabayashi et al., 2010a, Li et al., 2008). However, rat ESCs are less robust than their mouse counterparts and demand expert handling to maintain robust growth and capacity for germline transmission (Blair et al., 2011), especially after clonal selection required for gene targeting (Hirabayashi et al., 2010b, Hirabayashi et al., 2013, Hirabayashi et al., 2014, Meek et al., 2010, Men et al., 2012, Men and Bryda, 2013, Tong et al., 2010). These technical difficulties have hindered the widespread adoption of rat ESC transgenesis.

Meanwhile, the development of the CRISPR/Cas9 system (Cho et al., 2013, Cong et al., 2013, Hwang et al., 2013, Ma et al., 2014, Mali et al., 2013, Shen et al., 2013, Wang et al., 2013, Yang et al., 2013) has enabled rat genome editing via direct injection of one-cell embryos (Kim and Kim, 2014, Li et al., 2013a, Li et al., 2013b, Ma et al., 2014, Shao et al., 2014). The injected endonuclease is targeted to a specific DNA sequence by guide RNAs (gRNAs) and introduces double-strand breaks, which can be repaired by non-homologous end-joining (NHEJ) (Garneau et al., 2010, Lieber, 2010, Marraffini and Sontheimer, 2010). Error-prone NHEJ generally introduces small indels at the cleavage site to generate mutation in one or both alleles of the target sequence. Several knockout rats have been generated using this method (Li et al., 2013a, Li et al., 2013b). More recently, insertion of large DNA fragments at target loci has been achieved using single-stranded oligodeoxynucleotides (ssODNs) together with CRISPR/Cas9 (Chen et al., 2011, Storici et al., 2006, Yoshimi et al., 2014, Yoshimi et al., 2016). However, targeting efficiency varies unpredictably between different loci and according to the size of the insert. Moreover, both methods are inefficient and require injections of large numbers of embryos with associated maintenance of substantial numbers of animals. Furthermore, first-generation animals are generally mosaic, necessitating additional breeding and genotyping. Therefore, this approach does not provide the most efficient use of animals consistent with the 3R principles of reduction, refinement, and replacement. CRISPR/Cas9-mediated gene editing has also been applied in spermatogonial stem cells to create knockout rats (Chapman et al., 2015). Germline genome editing can avoid the production of mosaic mutant progeny (Brinster and Avarbock, 1994). However, homologous recombination has yet to be demonstrated, which limits applications.

Here, we tested whether CRISPR/Cas9 technology can be applied in rat ESCs both for *in vitro* studies and for generation of rats with targeted genomic insertions.

## Results

### Rat Embryonic Stem Cell Derivation and Culture

The culture conditions for rat ESCs were previously adjusted to reduce spontaneous differentiation by lowering the concentration of the glycogen synthase kinase-3 (GSK3) inhibitor CHIR99021 (CH) (Chen et al., 2013, Meek et al., 2013). However, even under these culture conditions, termed t2iL (see Experimental Procedures), rat ESCs still exhibit unreliable attachment to feeders, inconsistent growth rate and viability during routine passaging, sporadic differentiation, and a tendency to become tetraploid. These issues pose particular concern during the stringent clonal selection and expansion required for gene targeting. Therefore, we assessed several parameters during derivation of new ESC lines from Dark Agouti rats in t2iL. Conditions tested were: addition of the PKC inhibitor Gö6983 (Rajendran et al., 2013); addition of vitamin C (250 μM) (Esteban et al., 2010); use of Rho-associated kinase inhibitor Y-27632 (Watanabe et al., 2007); substitution of DMEM/F12 with lipid-rich advanced DMEM/F12; reduced oxygen atmosphere. We found that establishment of cell lines was most reliable using advanced DMEM/F12 in the base N2B27 formulation (Ying et al., 2003) together with t2iL, and with addition of Y-27632 in 5% O_2._ We selected one of the newly derived female ESC lines, DAC27, for use in subsequent experiments.

We first re-tested the effect of the empirical culture modifications on colony formation from single DAC27 cells. Advanced DMEM/F12 and reduced oxygen gave modest but additive improvements (Figure S1). Addition of Rho-associated kinase inhibitor Y-27632 (Watanabe et al., 2007) had a more substantial effect. The combination of aDMEM/F12, 5% O_2_ and Y-27632 gave a colony-forming efficiency of around 80% and moreover made routine passaging more consistent. We therefore incorporated all three modifications into the culture system for targeted genome modification.

### Targeted Mutation of *Lef1*

**Figure 1:**
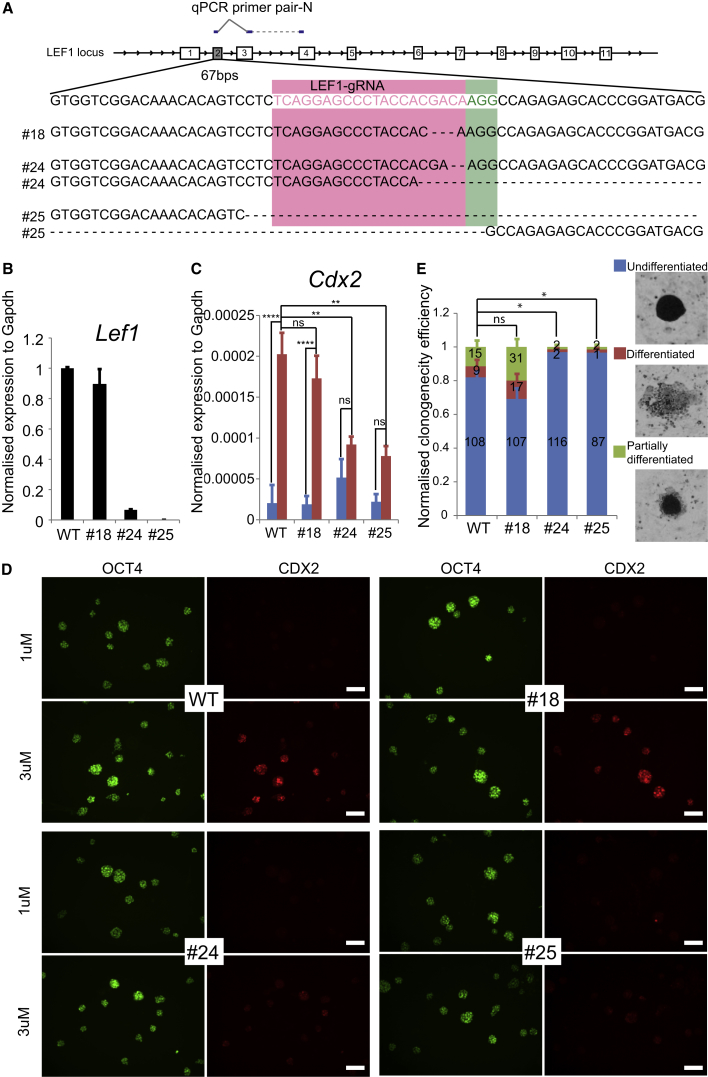
Generation and Characterization of *Lef1* Knockout rESCs (A) Design for CRISPR/Cas9-targeted mutation of *Lef1* exon2 and sequence of targeted clones. (B) Expression of *Lef1* transcript in parental and *Lef1* mutant rat ESCs assayed by RT-qPCR. Error bars represent the SD from three technical replicates. (C) Expression level of *Cdx2* transcript in response to CH at 1 μM or 3 μM. Error bars represent the SD of three independent experiments. Expression was normalized to *Gapdh*. ANOVA statistical analysis indicates the effect of CH significant (p < 0.0001) in wild-type (WT) and clone 18 but not in LEF1 mutant clones (clone 24, p = 0.4711; clone 25, p = 0.1567; ns, not significant; ^∗∗^p < 0.01, ^∗∗∗∗^p < 0.0001). (D) Fluorescent immunostaining of CDX2 and OCT4. Scale bars represent 100 μm. (E) Colony-forming assay in the presence of 3 μM CH. Undifferentiated, partially differentiated, and differentiated colonies were calculated relative to the total number of colonies counted for each line. Error bars represent SD from three biological replicates. ^∗^p < 0.001 (ANOVA).

Expression of the canonical Wnt signaling effector *Lef1* has been proposed to underlie the hypersensitivity of rat ESCs to the GSK3 inhibitor CH (Chen et al., 2013). We therefore chose the *Lef1* gene to test the applicability of CRISPR/Cas9 for targeted gene mutation in rat ESCs. A gRNA was designed using the CRISPR Design tool (http://crispr.mit.edu/) to target the second exon of *Lef1* (Figure 1A).

For *Lef1* targeting, 1 × 10^6^ rat ESCs were transfected using Lipofectamine 2000 with 1.2 μg of expression plasmid containing gRNA and Cas9-2A-GFP. Eight hours post transfection, cells were replated onto new feeders in fresh medium. Twenty-four hours after replating, GFP-positive cells were sorted by flow cytometry into 10 cm culture dishes at a density of 10,000 cells per dish. Fifteen milliliters of medium was added into each dish, and no medium change was required thereafter. Five days later, individual colonies were picked, plated into duplicate 96-wells, and expanded briefly before genotyping one of the duplicates.

Genomic PCR followed by gel electrophoresis indicated that 5 of 38 (13%) expanded cultures had an overt deletion in one or both alleles of *Lef1* (Figure S2A). We selected three clones with distinct gPCR products: cl18, no overt size change; cl24, one smaller band; cl25, no wild-type band. We subcloned 24 and 25 and repeated the gPCR screen to eliminate the possibility of mixed colonies from the primary plating. We then sequenced the genomic region spanning *Lef1* exon2. Clone 18 had an in-frame deletion of 3 bp, with no wild-type sequence. Clone 24 had a 2 bp frameshift mutation in one allele and a deletion of 124 bp in the other allele. Clone 25 had deletions of 173 and 506 bp (Figure 1A).

To assess whether *Lef1* expression was indeed disrupted in these three clones, we designed primers flanking the gRNA recognition site and performed RT-qPCR analysis (Figure 1B). Clone 18 yielded a PCR product in similar amount to parental cells. Consistent with sequencing results, clone 25 yielded no detectable product. Analysis of clone 24, on the other hand, indicated a residual level of transcript. This could be due to incomplete nonsense mediated mRNA decay of the frameshifted transcript. We examined LEF1 protein expression by immunocytochemistry using a monoclonal antibody that detects an epitope downstream of the deleted region encoded by exon 2. Strong staining was observed in parental and clone 18 cells, while clone 24 and clone 25 cells were unstained (Figure S2B). Collectively, these data indicate that clones 24 and 25 are null mutants lacking LEF1.

We examined the phenotypic consequence of loss of LEF1. In standard 2iLIF medium containing 3 μM CH (Ying et al., 2008), expression of Wnt targets related to differentiation, such as CDX2, is appreciable in rat ESCs (Chen et al., 2013, Meek et al., 2013). To investigate whether inactivating *Lef1* could alleviate the hypersensitivity of rat ESCs to GSK3 inhibition, we first measured the induction of *Cdx2* by RT-qPCR. In parental cells, the expression of *Cdx2* increased more than 6-fold when CH concentration was raised from 1 μM to 3 μM. Clone 18 cells showed a similar response to CH, suggesting that loss of a single amino acid has a minor effect on LEF1 function. In contrast, the *Cdx2* response to CH was reduced in clones 24 and 25 (Figure 1C). Expression of CDX2 protein was also markedly attenuated in these two *Lef1* mutant clones, as shown by immunofluorescence staining (Figure 1D).

To assess whether loss of *Lef1* had an impact on the self-renewal of rat ESCs, we performed colony-forming assays in the presence of 3 μM CH. Colonies were stained for alkaline phosphatase and scored for level of differentiation, categorized as undifferentiated, partially differentiated, or differentiated. A representative image of each category is shown in Figure 1E. As previously reported (Chen et al., 2013), differentiation was overt in around 20% of parental rat ESCs cultured in 3 μM CH. This was also apparent in clone 18. In contrast, in clone 24 and clone 25 mutants, fewer than 5% of colonies contained differentiated cells (Figure 1E). We also observed that clones 24 and 25 could be propagated readily in standard 2iL with no evident detriment compared with t2iL, in contrast to parental or clone 18 cells. These results are consistent with LEF1 mediating differentiation sensitivity of rat ESCs to GSK3β inhibition.

### Generation of a Non-disruptive *Sox10* Knockin Reporter

Based on the proof of principle of genome editing in rat ESCs, we sought to generate a targeted knockin modification via CRISPR/Cas9-facilitated homologous recombination. We chose the *Sox10* gene in order to create a reporter rat of value to the developmental biology and neuroscience communities. *Sox10* is a member of the Sry-related HMG box (Sox) family of transcription factors. It is expressed throughout the developing neural crest (Kelsh, 2006) and in all oligodendroglial lineage cells (Stolt et al., 2002). One particular attraction of a *Sox10* reporter is as a tool for visualizing and isolating precursor and mature oligodendrocytes from postnatal animals. Indeed several transgenic mouse lines have been created using the *Sox10* promoter (Kessaris et al., 2006, Rinholm et al., 2011, Shibata et al., 2010, Simon et al., 2012). However, rats in which oligodendrocyte lineage cells are specifically labeled would be a valuable resource due to the relative ease of surgical procedures (Iannaccone and Jacob, 2009) and superiority of demyelinating lesions models in the rat (Woodruff and Franklin, 1999), combined with their greater suitability for learning and cognition assays (Iannaccone and Jacob, 2009).

**Figure 2:**
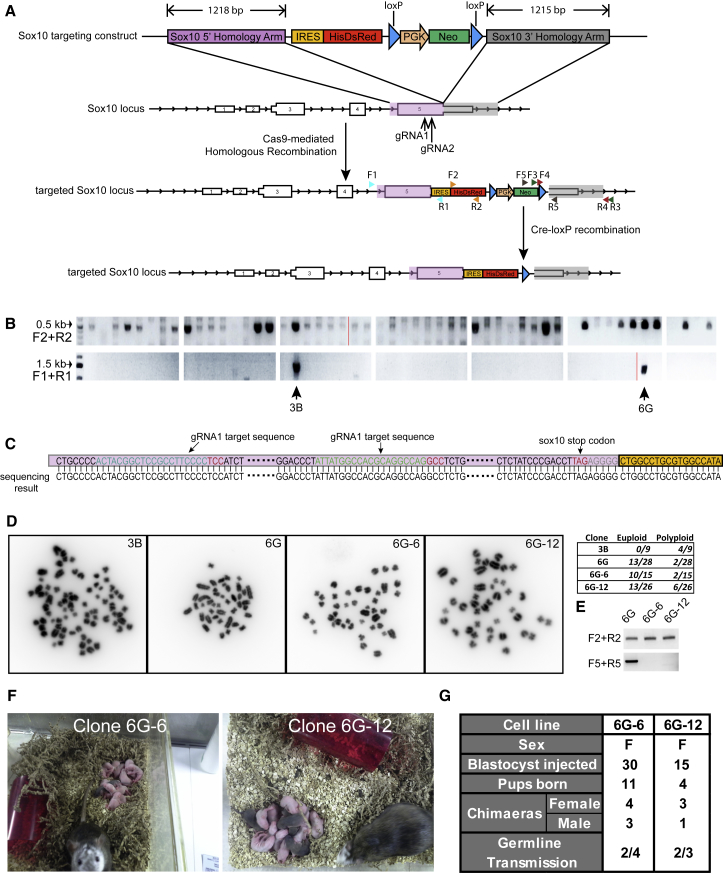
Generation of Sox10-dsRed Reporter Transgenic Rat (A) Design of *Sox10* targeting. (B) Screen for targeting by genomic PCR. (C) Genomic sequence around gRNA recognition sites in clone 6G cells. (D) Representative images and chromosome counts of metaphase spreads in Sox10 targeted clones. (E) Genomic PCR assay for excision of PGK-neo^R^ selection cassette (F) Chimeras and germline F1 pups following injection of Dark Agouti ESCs 6G-6 and 6G-12 into SD (albino) blastocysts. (G) Summary of chimeras and test breeding.

In common with several other *Sox* gene family members, *Sox10* is haploinsufficient (Britsch et al., 2001, Paratore et al., 2002). It is therefore essential to avoid disruption of endogenous SOX10 expression in any knockin reporter. We designed a construct to insert an internal ribosome entry site (IRES) coupled to a red fluorescent protein (HisDsRed) coding sequence into the 3′ UTR, leaving the *Sox10* gene structure intact (Figure 2A). dsRed is fused to a histidinol resistance enzyme, allowing the potential option for drug selection of *Sox10*-expressing cells if required. The insertion site was selected 5 bp downstream of the stop codon. Fragments of approximately 1.2 kb of genomic sequence were amplified by genomic PCR to generate 5′ and 3′ homology arms. *Sox10* is not expressed in ESCs; therefore, positive selection was provided by a PGK-Neo cassette flanked by *Loxp* sites.

We designed two gRNAs with recognition sites close to the designated insertion site in the 3′ UTR. We introduced the gRNAs together with the *Sox10-IRES-HisDsRed* targeting vector and *Cas9* nickase plasmid into 1 × 10^6^ DAC27 rat ESCs via lipofection. Use of Cas9 nickase is reported to increase the ratio of homology-directed repair to NHEJ and reduce off-target genome disruption (Cong et al., 2013, Mali et al., 2013, Rong et al., 2014). Transfected cells were replated after 8 hr into 4 × 10 cm dishes on feeders overlaid with Matrigel. After 24 hr, G418 (300 μg/mL) selection was applied. Colonies were picked after 7 days and expanded without further selection in duplicate for genotyping.

Two of 52 picked colonies, 3B and 6G, yielded a band of the expected size (∼1.4 kb) for homologous recombination detected by genomic PCR using a primer pair flanking the 5′ homology arm (Figure 2B). These two clones were validated further using primers to amplify the reporter region and the 3′ homology arm (Figure S3). To check whether CRISPR/Cas9 editing had created undesired mutations close to the gRNA recognition sites, we sequenced these genomic regions. No mutations were detected in either clone (Figure 2C).

We assessed the chromosome complement of the two clones by metaphase analysis. Clone 3B was comprised of hyperdiploid and tetraploid cells (Figure 2D) and was discarded. Clone 6G had a proportion of tetraploid cells, but 13 out of 28 spreads examined (46%) had a euploid count of 42 chromosomes (Figure 2D). This clone was therefore chosen to proceed to the next step. Cells were transfected with a Cre recombinase expression plasmid and subsequently plated at low density (10,000 cells per 10 cm dish) for sub-cloning. Individual colonies were picked and split into duplicate wells of a 96-well plate and cultured with or without G418. Loss of resistance to the antibiotic indicated excision of the PGK-Neo cassette.

Chromosome counts were again checked by metaphase analysis. Two of 12 clones, clone 6G-6 and clone 6G-12, contained at least 50% euploid cells (Figure 2D). Genomic PCR confirmed the absence of PGKneo^R^ in both clones (Figure 2E). They were expanded briefly before injection into blastocysts of the albino SD strain. Coat color chimeras were obtained in both cases (Figure 2F). Female chimeras were test mated to SD males and from each clone, two animals proved to be germline competent in the first litter (Figures 2F and 2G). These data demonstrate that rat ESCs maintained using aN2B27-t2iLY in 5%O_2_ can maintain full competence after two rounds of genetic engineering and clonal selection.

### *Sox10* Reporter Characterization

**Figure 3:**
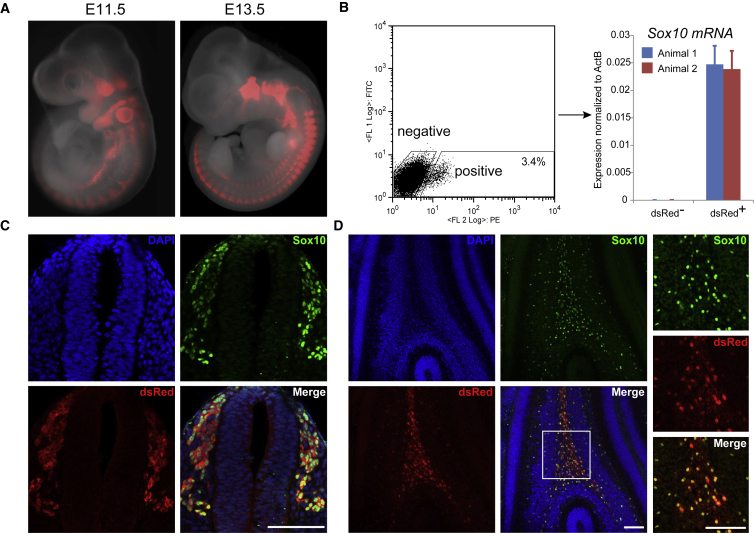
Characterization of Sox10-dsRed Reporter Transgenic Rat (A) Fluorescent images of E11.5 and E13.5 embryos. (B) Live sorting of dsRed^−^ and dsRed^+^ cells from E11.5 embryos followed by RT-qPCR for *Sox10* transcript. Error bars represent the SD from three technical replicates. (C) Immunostaining for dsRed and SOX10 on 100 μm cross-sections of the spinal cord region at E13.5. Scale bars represent 100 μm. (D) Immunostaining of 100 μm cross-sections of P7 newborn rat cerebellum. The white boxed area is shown in higher magnification in the right panel. Scale bars represent 100 μm.

Germline offspring from both knockin clones were bred with SD animals to establish transgenic lines. Heterozygous outcross matings were employed to characterize reporter expression. We evaluated the pattern of dsRed fluorescence at two developmental stages. At embryonic day 11.5 (E11.5), dsRed signal was readily detected in neural crest cell derivatives and the otic placode (Figure 3A), consistent with the pattern of SOX10 expression during embryonic development (Breuskin et al., 2009, Breuskin et al., 2010). At E13.5, fluorescence signals were prominent in dorsal root ganglia (DRG) and trigeminal ganglia, as expected.

We dissociated E11.5 embryos into single cells and sorted dsRed-positive and -negative populations by flow cytometry. Approximately 3.5% live cells were positive for dsRed fluorescence. The positive and negative populations were analyzed for *Sox10* mRNA by RT-qPCR (Figure 3B). *Sox10* transcript was only detected in the dsRed-positive population, indicating faithful reporting of endogenous *Sox10* transcripts. We carried out double immunofluorescent staining for SOX10 and dsRed proteins on sections from E13.5 embryos and P7 newborn rat brain cerebellum. As shown in Figures 3C and 3D, respectively, SOX10 antibody stained nuclei in the DRG region of E13.5 embryos and the cerebellum of P7 neonates. dsRed was detected in the cytoplasm of the same subset of cells. Notably, we did not observe expression of dsRed without co-expression of SOX10.

Following outcrossing of F1 animals, we set up intercross matings to acquire homozygotes. Homozygous animals were obtained from both clones. We euthanized a homozygote at 14 weeks and prepared brain sections. dsRed-positive cells were evenly distributed across the cortex, corpus callosum, and sub-cortical regions (Figure S4A). Immunostaining for myelin basic protein indicated a normal pattern of myelin deposition in white and gray matter (Figures S4B–S4D). Co-expression of dsRed was detected in Olig2-positive cells and in NG2-positive oligodendrocyte progenitors (Figures S4E–S4H).

Mice and humans heterozygous for *Sox10* loss of function mutations display overt phenotypes: abnormal pigmentation and megacolon in mice; Hirschsprung disease in humans (Britsch et al., 2001, Paratore et al., 2002). Such haploinsufficiency implies that SOX10 protein dosage is critical. In contrast, we have observed no abnormalities in multiple heterozygous and homozygous animals. We surmise that *Sox10::dsRed* knockin rats express functional SOX10 at physiologically sufficient levels. We cryopreserved embryos derived from clone 6G-12 and have deposited live rats with the Rat Resource & Research Center.

### Whole-Cell Patch-Clamp Recording from dsRed-Positive Oligodendrocyte Lineage Cells

**Figure 4:**
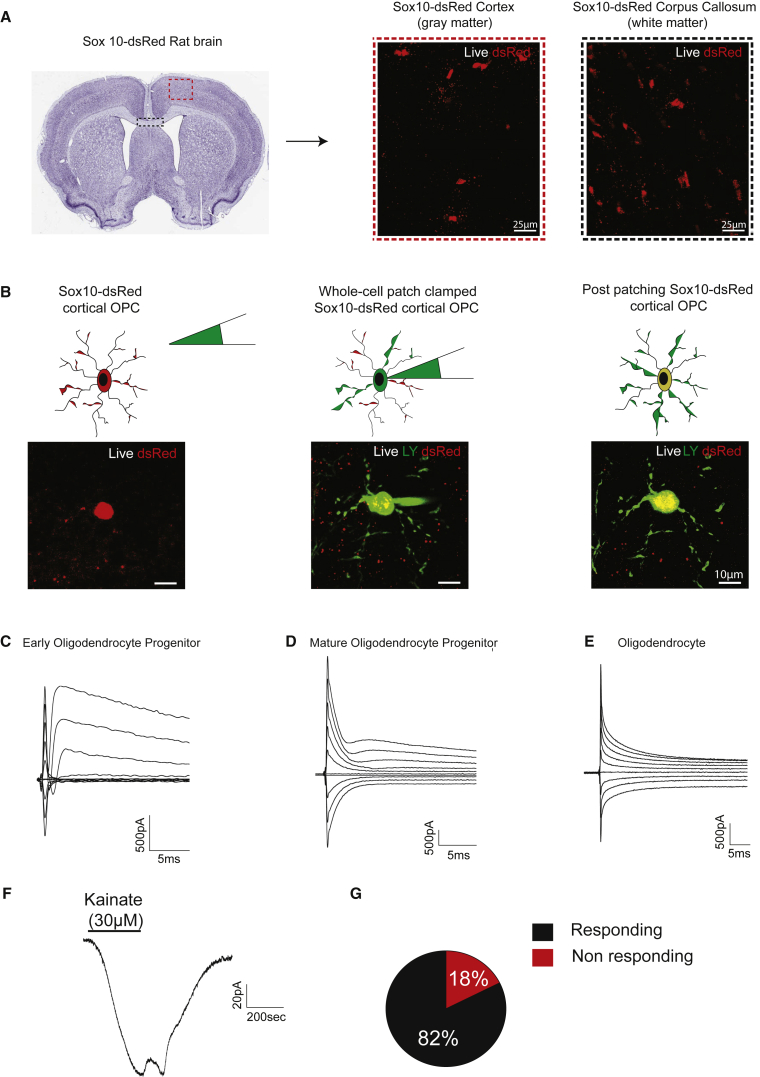
Imaging and and Patch-Clamp Characterization of Sox10-dsRed-Positive Cells in Brain Slices (A) Representation of a cortical Sox10-dsRed rat coronal brain slice. Magnification of red dashed insert shows the live detection of dsRed^+^ oligodendrocyte lineage cells in the rat cortex. Magnification of the black dashed insert represents the live detection of dsRed^+^ oligodendrocyte lineage cells in the rat white matter (corpus callosum). (B–E) Schematic and imaging representation of live dsRed detection with simultaneous dye-filling with lucifer yellow (LY) of an oligodendrocyte lineage cell during whole-cell patch-clamp recordings (B). Scale bars represent 10 μm. Characteristic I-V and voltage-gated Na_v_ expression levels in dsRed-Sox10^+^ (C) early oligodendrocyte progenitor cells, (D) mature oligodendrocyte progenitors, and (E) oligodendrocytes. (F and G) Representative response of dsRed-Sox10^+^ rat oligodendrocyte lineage cells to kainate (30 μM) (F) and the percentage of responsive and non-responsive DsRed-Sox10^+^ rat oligodendrocyte lineage cells to kainate (G). Total number of cells used for (G), n = 17 from N = 4 separate biological replicates.

We examined whether this reporter rat can facilitate the study of oligodendroglial cells. First, we checked whether a dsRed signal can be detected in living cells in postnatal rat brain. In freshly prepared coronal brain slices, we identified dsRed^+^ cells by fluorescence microscopy in both cortex (gray matter) and corpus callosum (white matter). Positive cells displayed morphology of oligodendrocyte lineage cells (Figure 4A). All stages of oligodendroglia are expected to be labeled by *Sox10*:*dsRed*. To confirm this, fluorescent cells were selected for whole-cell voltage-clamp recordings. Cells of different lineage stages were identified based on morphology assessed through live-cell imaging. Once cells were patched in whole-cell mode, lineage stage was further evaluated by morphology through additional lucifer yellow dye labeling. The current response evoked by a 10 mV pulse (150 ms duration) was used to determine the decay constant and input resistance, and voltage-current membrane properties were used to analyze voltage-gated sodium and potassium currents. Cartoons and live images of cortical oligodendrocyte progenitor cells are shown in Figure 4B. Representative recordings of early oligodendrocyte progenitors, mature oligodendrocyte progenitors, and fully differentiated oligodendrocyte are shown in Figures 4C–4E. The electrophysiological properties are consistent with the morphological assessments of maturation stage.

Oligodendrocyte lineage cells express glutamate receptors including kainate receptor (Verkhratsky and Steinhauser, 2000) and respond to kainate stimulation (Figure 4F). Therefore, we also measured the kainate response in dsRed^+^ cells. More than 80% showed a response, further indicating that *Sox10*:*dsRed* identifies functional oligodendrocytes in the brain.

## Discussion

Here, we have documented the application of CRISPR/Cas9-mediated genome editing in rat ESCs and demonstrated the utility for generation of *in vitro* and *in vivo* models. Incremental refinements of rat ESC culture conditions conferred more consistent growth and clonogenicity, facilitating recovery of clones after genetic manipulation. In addition, we took two further measures to maximize the probability of germline transmission: first, we used rat ESCs at low passages; second, we selected diploid clones after each round of clonal selection. Combined with use of the CRISPR/Cas9 system, these refinements increase the practicality of using rat ESCs for gene targeting. Although the incidence of *Sox10* knockin was only 4% of stable transfectants, the construct used lacked any negative selection cassette, commonly included to enrich for homologous recombinants. Ease of vector preparation due to shorter homology arms is an advantage of using Cas9 compared with conventional homologous recombination. More importantly, both of the targeted sub-clones selected for blastocyst injection gave high-contribution chimerism and germline transmission.

The creation of *Lef1* mutant rat ESCs allowed examination of the significance of *Lef1* downstream of GSK3 inhibition. This analysis provided further evidence that *Lef1* contributes to destabilization of self-renewal via induction of lineage specification genes downstream (Chen et al., 2013, Meek et al., 2013). These results may explain why the optimal concentration of GSK3 inhibitor CH is only 1 μM compared with 3 μM for mouse ESCs. Interestingly, human naive pluripotent stem cells also express *Lef1* and show a similar requirement for titrated CH (Takashima et al., 2014).

Rat ESCs can be exploited as an *in vitro* differentiation system, complementary to mouse and human pluripotent stem cells. Knockin reporters are extremely useful tools in this context. For example, the *Sox10* reporter generated here could be exploited for monitoring differentiation into neural crest or oligodendroglia, and for purifying desired cell populations.

ESC-mediated genome engineering has been transformative in mouse genetics and now provides similar opportunities in the rat, which in several areas of physiology and neuroscience has considerable advantages over the mouse as a model species. The *Sox10::dsRed* rat model generated here can facilitate study of neuron-oligodendrocyte interactions and remyelination. Importantly, the rat is preferred to the mouse in this context. Notably rats are used for the cerebellar caudal peduncle (CCP) ethidium bromide model of myelin regeneration (Goudarzvand et al., 2016, Woodruff and Franklin, 1999). The CCP is one of the few fully myelinated tracts in the brain and is often affected by demyelinating disease, such as multiple sclerosis (Preziosa et al., 2014). Rats are used for this lesion because the CCP is not accessible to surgery in mice. More generally, neuropharmacology, cellular distribution of neurotransmitter receptors, and neurotransmitter receptor structure are more similar between humans and rats (Hirst et al., 2003). The *Sox10* reporter rat may also be useful in investigations of white matter plasticity, taking advantage of the repertoire of behavioral assays available for rats.

In conclusion, these findings demonstrate that CRISPR/Cas9 methodology can readily be implemented in rat ESCs. Genome editing in rat ESCs constitutes a powerful system for comparative molecular genetic dissection of *in vitro* pluripotent stem cell biology. More broadly, the capacity for germline transmission provides a platform for generating advanced animal models in this important species for biomedical research.

## Experimental Procedures

### Production of Rat Chimeras

Blastocyst microinjection was carried out as previously described (Blair et al., 2012) using host blastocysts from the albino Sprague-Dawley strain. Chimeras were identified by mixed coat color. All animal studies were approved by the UK Home Office and carried out in a designated facility.

### Cell Culture

Rat ESCs were derived from E4.5 blastocysts from the Dark Agouti strain and maintained on γ-irradiated mouse embryo fibroblasts in aN2B27 medium supplemented with t2iL + Y, consisting of MEK inhibitor PD0325901 (1 μM), GSK3 inhibitor CHIR99021 (1 μM), human recombinant leukemia inhibitory factor (10 ng/mL, prepared in house) and Rho-associated kinase inhibitor, Y-27632 (5 μM). Cultures were maintained in a humidified incubator at 37°C in 7% CO_2_ and 5% O_2_. To prepare aN2B27, we used advanced DMEM/F12 (Gibco, catalog no. 12491015). Rat ESCs were routinely passaged by dissociation into single cells with TrypLE Express every 48 hr and replated at a split ratio between 1:4 and 1:6. Sufficient culture medium was added when seeding the plates that no change was required until the next passage.

### Gene Targeting via CRISPR/Cas9

A newly derived rat ESC line, DAC27, was used at passage 8 for gene targeting experiments. gRNAs were designed using the CRISPR Design tool (http://crispr.mit.edu/) to target the desired region (Table S1). For *Lef1* targeting, 1 × 10^6^ rat ESCs maintained in aN2B27 (t2iL + Y, 5% O_2_) were transfected using Lipofectamine 2000 with 1.2 μg of expression plasmid containing gRNA, Cas9, and GFP (pSpCas9(BB)-2A-GFP (PX458), a gift from Feng Zhang; Addgene plasmid no. 48138) (Ran et al., 2013). Eight hours post transfection, cells were replated onto new feeders in fresh medium. Twenty-four hours after replating, GFP-positive cells were sorted by fluorescence-activated cell sorting onto 10 cm culture dishes at a density of 10,000 cells per dish. Fifteen milliliters of medium was added into each dish and no medium change was required thereafter. Five days after seeding, individual colonies were picked, expanded briefly, and screened using genomic PCR. For generation of *Sox10* knockin, 1 × 10^6^ cells were transfected with 1.2 μg of gRNA plasmid, 1.2 μg of Cas9 nickase plasmid, and 1.2 μg of targeting vector. We used circular plasmids to minimize random integration. Eight hours post transfection, cells were replated onto 4 × 10 cm culture dishes and G418 selection commenced 24 hr later. Medium was replaced every 24 hr for the first 4 days and every 48 hr thereafter. To ensure robust attachment of rat ESCs during selection, a thin layer of Matrigel (BD Matrigel, 1:240 dilution in MEF medium) was applied to the MEF feeder layer 24 hr before rat ESC seeding. After 1 week of selection, individual colonies were picked, expanded briefly, and screened by genomic PCR. To confirm *Lef1* targeted clones, multiple sets of genotyping primers were used to analyze up to 2.2 kb around the gRNA recognition site, and genomic PCR products were sequenced for mutations and deletions (Table S3). To confirm *Sox10* targeted clones, genomic PCR product amplified using *Sox10 set1 (5HA)* primers was inserted into TA clones and sequenced using M13 forward and reverse primers. The region close to the *Sox10* gRNA recognition site was also sequenced using customized primer to confirm the absence of mutations.

### Gene Expression Analysis by Real-Time qPCR

Total RNA was isolated using the RNeasy Kit (QIAGEN) and cDNA prepared using SuperScriptIII (Invitrogen) and 3′RACE adapter primers. Primers and probes used for real-time PCR are listed in Table S2.

### Chromosome Analysis

Cells were treated for 2.5 hr with colcemide (Gibco, 1:100 dilution) 24 hr after passaging. Metaphase chromosome spreads were prepared and imaged at 63×. Chromosomes in discrete spreads were counted.

### Immunofluorescence Cell Staining

Cells were fixed with 4% paraformaldehyde in PBS (pH 7.0) for 30 min at room temperature. Subsequently, cells were washed twice with PBST (0.1% Triton X-100 [Sigma] in 1× PBS) and then with blocking solution (4% donkey serum in PBST). Primary antibody solution was prepared by diluting antibody in blocking solution at the concentration listed in Table S4. Cells were incubated with the primary antibody at room temperature for 2 hr or at 4°C overnight, followed by three washes with PBS containing 0.1% Tween 20 prior to incubation with the secondary antibodies at room temperature for 1 hr. After nuclear staining with DAPI (Invitrogen), stained cells were detected by fluorescence microscopy.

### Fluorescence-Activated Cell Sorting

Fluorescent E11.5 embryos were cut into small pieces before incubating in TrypLE Express enzyme for 15 min at room temperature. Digested tissue was triturated using a p1000 pipette. Enzyme was inactivated and diluted with serum containing wash buffer. Larger debris was removed with 100 μm cell strainers before re-suspension in 1 mL of PBS containing 2% BSA for sorting using a Bio-Rad S3 cell sorter.

### Immunostaining of Rat Brain

*Sox10::dsRed* rats at 14 weeks were perfused with 4% paraformaldehyde, and coronal sections cut on a vibratome (100 mm). Fixed slices were incubated for 5 hr in 0.5% Triton X-100, 10% goat serum in PBS at 21°C, then with primary antibody at 21°C overnight, and then for 5 hr at 21°C with secondary antibody. Cryostat sections were incubated for 1 hr in 0.1% Triton X-100, 10% goat serum in PBS at 21°C, then with primary antibody at 4°C overnight, and then for 1 hr at 21°C with secondary antibody. Primary antibodies were: rabbit or mouse RFP (Abcam, 1:100), mouse NG2 (Millipore, 1:100), rabbit Olig2 (Millipore, 1:300), rabbit MBP (Sigma, 1:100). Secondary antibodies were goat anti-rabbit IgG and anti-mouse IgG (Life Technologies, 1:1,000). DAPI (Sigma) was used to label nuclei (10 min, 1 μg/mL).

### Electrophysiology

Parasagittal cerebellar slices (225 μm) were prepared from P3-10 *Sox10*::*dsRed* rats using a vibrating blade microtome (Leica VT1200S). After dissection, the brain was placed in a cooled (∼1°C) oxygenated (95% O_2_/5% CO_2_) Krebs solution containing: 126 mM NaCl, 24 nM NaHCO_3_, 1 mM NaH_2_PO_4_, 2.5 mM KCl, 2.5 mM CaCl_2_, 2 mM MgCl_2_, 10 mM D-glucose (pH 7.4). Kynurenic acid was included to block glutamate receptors, which might be activated during the dissection procedure and cause cell damage. During experiments, slices were superfused with HEPES-buffered external solution containing: 144 mM NaCl, 2.5 mM KCl, 10 mM HEPES, 1 mM NaH_2_PO_4_, 2.5 mM CaCl_2_, 10 mM glucose, 0.1 mM glycine (to co-activate NMDA receptors), 0.005 mM strychnine (to block glycine receptors). pH was set to 7.4 with NaOH and the solution was permanently bubbled with 100% O_2_. Recording electrodes were filled with an internal solution comprising: 130 mM K-gluconate, 4 mM NaCl, 0.5 mM CaCl_2_, 10 mM HEPES, 10 mM BAPTA, 4 mM MgATP, 0.5 mM Na_2_GTP, 2 mM K-lucifer yellow, pH set to 7.3 with KOH; electrode resistance ranged from 5 to 9 MΩ. Series resistance was left uncompensated and averaged at 30 ± 1.5 MΩ. Electrode junction potential of −14 mV was compensated for. A Multiclamp 700B (Molecular Devices) was used for voltage-clamp data acquisition. Data were sampled at 50 kHz and filtered at 10 kHz using pClamp10.3 (Molecular Devices).

## Author Contributions

Y.C. performed and interpreted experiments; S.S. performed the patch-clamp recording; and S.A. performed live imaging of dsRed^+^ oligodendrocyte lineage cells and prepared the figure. R.T.K. designed and supervised the electrophysiology experiments and performed immunostaining of brain sections. A.S. designed and supervised the study and wrote the paper with Y.C.

## Supplemental Information

Document S1. Targeting Protocol, Figures S1–S4, and Tables S1–S4

Document S2. Article plus Supplemental Information
