# Supplementary material for: Gene Editing in Rat Embryonic Stem Cells to Produce In Vitro Models and In Vivo Reporters
Source: Stem Cell Reports. 2017 Oct 10;9(4):1262–74. doi: 10.1016/j.stemcr.2017.09.005 (PMC5639479; doi:10.1016/j.stemcr.2017.09.005)
Supplement: Document S1. Targeting Protocol, Figures S1–S4, and Tables S1–S4 [file mmc1.pdf]

Stem Cell Reports, Volume *9*

Supplemental Information

Gene Editing in Rat Embryonic Stem Cells to Produce *In Vitro* Models and *In Vivo* Reporters

Yaoyao Chen, Sonia Spitzer, Sylvia Agathou, Ragnhildur Thora Karadottir, and Austin Smith

Supplementary Figure 1

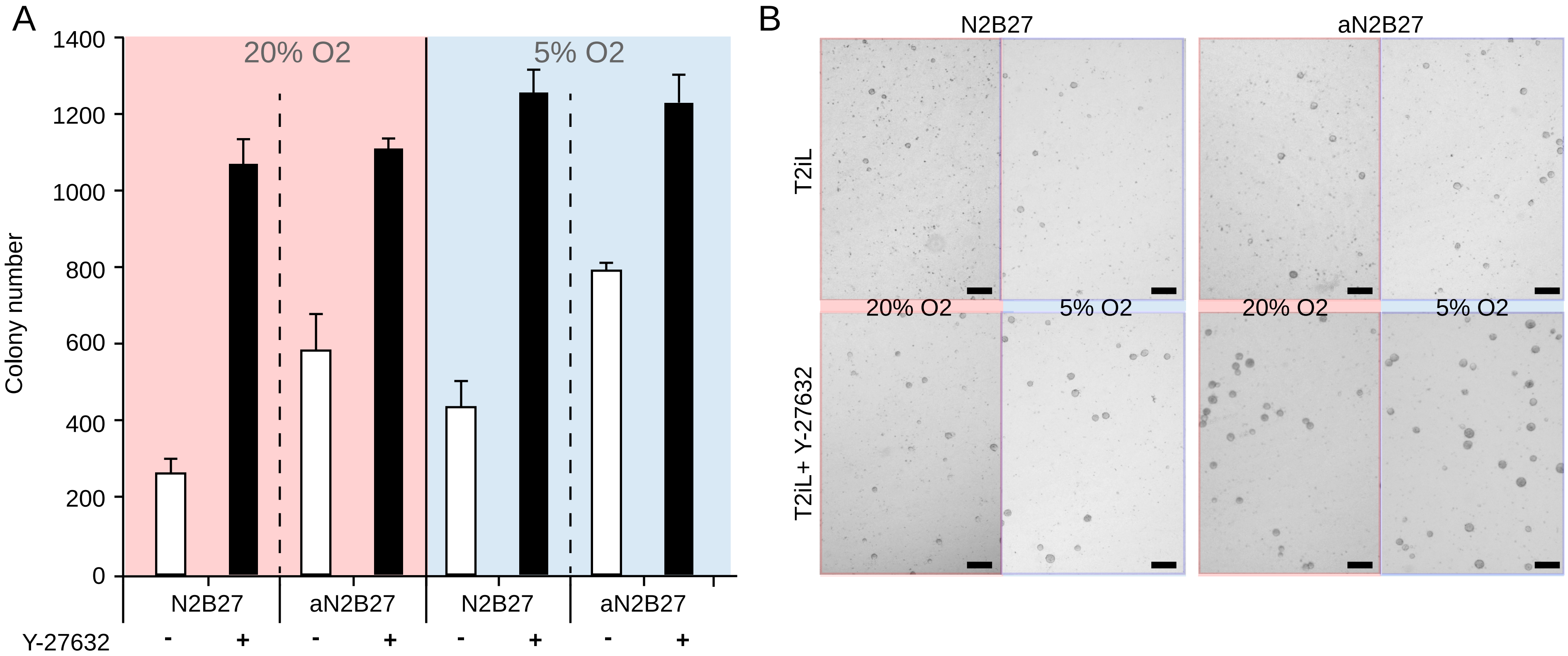

Supplementary Figure 2

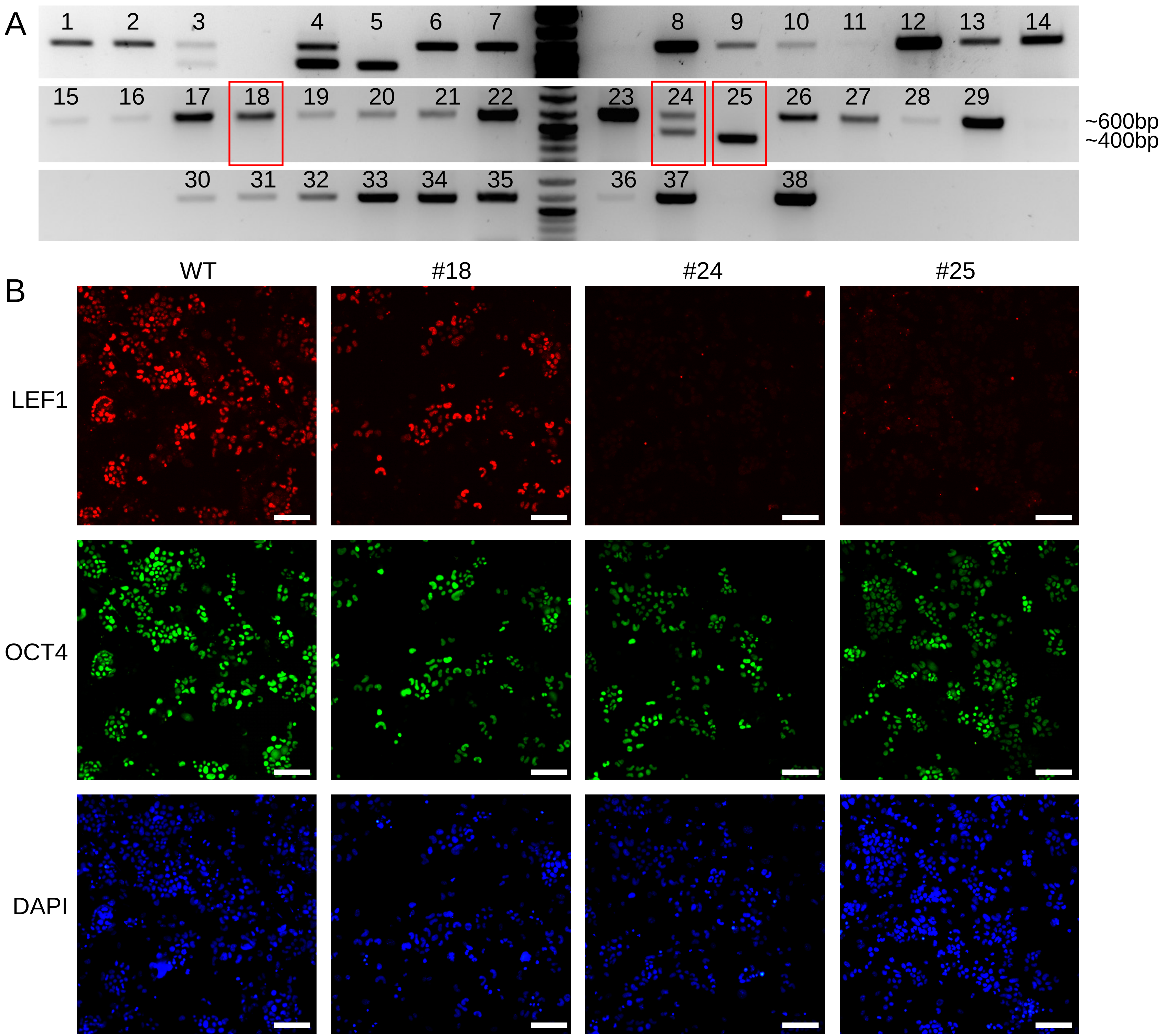

Supplementary Figure 3

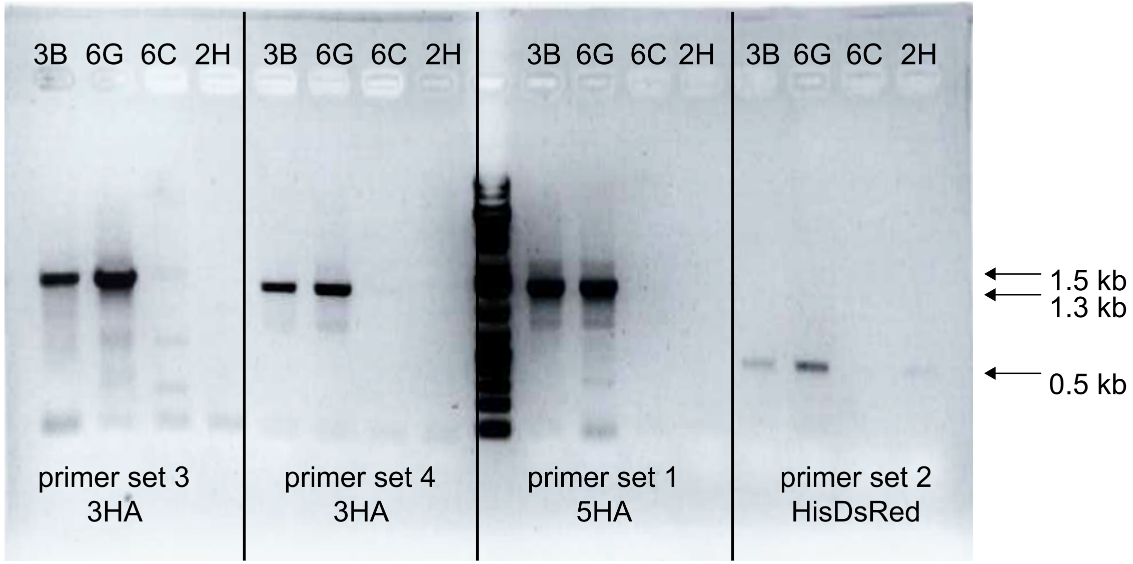

Supplementary Figure 4

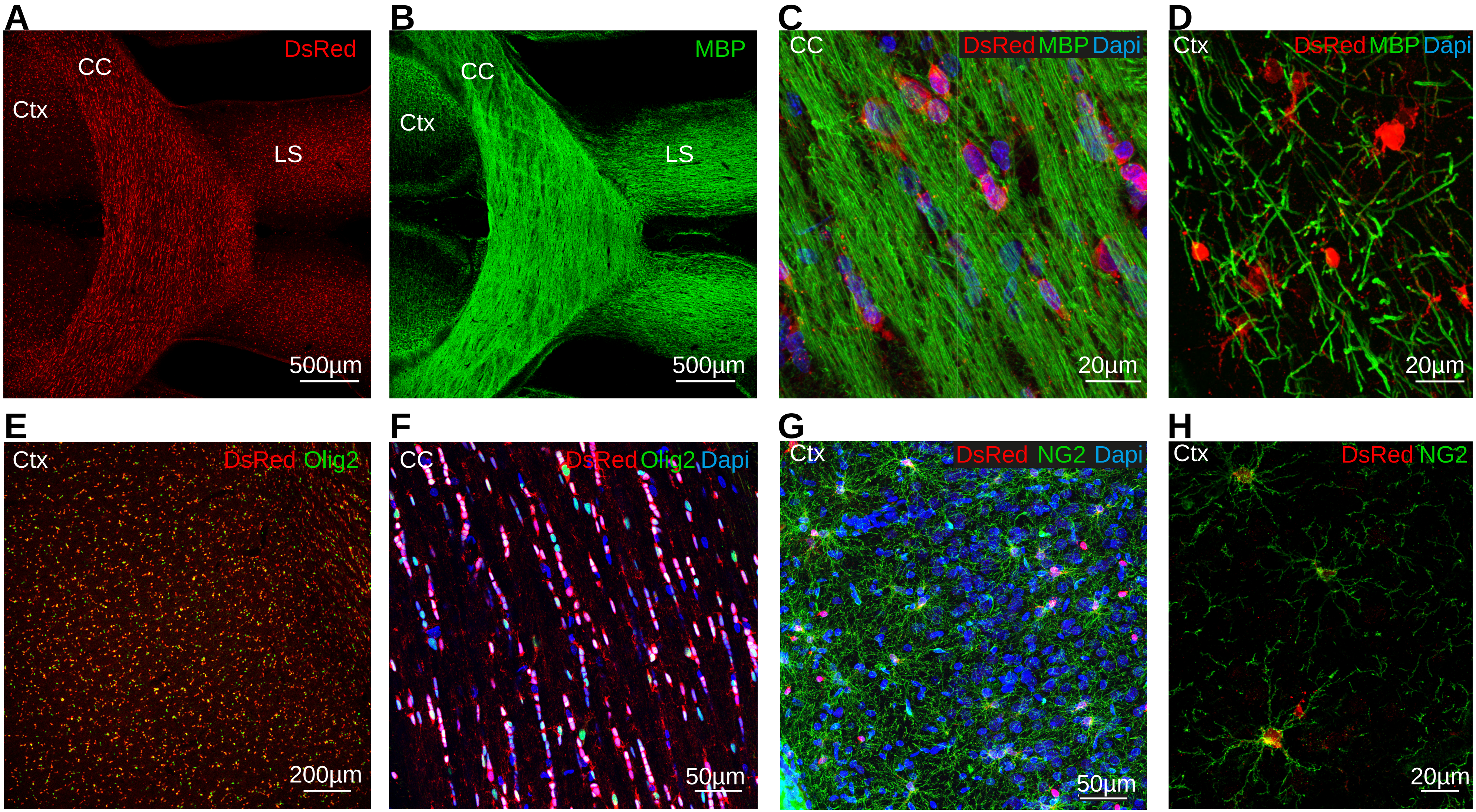

Supplementary Information

Figure S1: **Comparison of rat ES cell culture parameters**.

Related to first section of Results. (A) Colony formation in different culture conditions. For each condition, 1500 cells (per well) were seeded into 3 replicate wells in 12 well plates. The starting rat ES cell population (DAC27) was cultured in aN2B27 with Y-27632 in the presence of 5% O_2. Error bars represent SD of three biological replicates. (B) Representative images of rat ES cells in different culture conditions. Scale bars represent 200μm.

Figure S2: ***Lef1* knockout.**

Related to Figure 1. (A) Screen for *Lef1* mutation by genomic PCR. (B) Fluorescent co-staining of LEF1 and OCT4 in parental and *Lef1* mutant rES cells cultured for 24 hours on laminin coated black 24-well glass-bottom plates. Scale bars represent 100μm.

Figure S3: **Genotyping of *Sox10* targeted clones**

Related to Figure 2. Both clone 3B and 6G gave expected size bands when using indicated genotyping primer sets. Clone 6C and 2H were used as non-recombined controls.

Figure S4: **DsRed is co-expressed with oligodendrocyte lineage markers in adult rat brain**

Related to Figure 3. (A) Low magnification coronal section showing evenly distributed expression of *Sox10-DsRed* (red) in the cortex (Ctx), Corpus Callosum (CC) and subcortical areas (LS: Lateral Septal Nucleus). (B-D) Myelination appears normal in the *Sox10-DsRed* rats (Myelin Basic Protein: MBP; green, Cell nuclei: Dapi; blue, DsRed; Red), in both (C) white matter (CC) and (D) grey matter (Ctx –Layer 1) – Note that recombinant proteins are excluded from compact myelin. E. Cells expressing DsRed express the oligodendrocyte lineage marker Olig2 (Green), in both grey (E) and white matter (F). (G-H) Oligodendrocyte progenitor cells (NG2 positive cells, NG2; green) express DsRed. Scale bars are indicated on the figure.

Table S1. Guide RNA sequences

Gene	gRNA sequence
Sox10 gRNA1	CTGGCCTGCGTGGCCATAAT
Sox10 gRNA2	GACAAACACAGTCCTCTCAG
Lef1 gRNA	TCAGGAGCCCTACCACGACA

Table S2. Primers and probes for real-time PCR.

Gene	Forward primer sequence	Reverse primer sequence
Lef1	GACAAACACAGTCCTCTCAG	GCACTTTATTTGAGGTCCTCG

Gene	Company	Cat. No.
Gadph	Appliedbiosystem	4352338E
Cdx2	Appliedbiosystem	Rn00576694_m1 (Cat#:4331182)

Table S3. Primers for genomic PCR.

Gene	Forward primer sequence	Reverse primer sequence
Sox10 set1 (5HA)	gcctagcatgttctgctcgagagccc	cccctctaaggtcgggatagagt
Sox10 set2(HisDsRed)	gacaaacacagtcctctcag	gcactttatttgaggtcctcg
Sox10 set3 (3HA)	gcaaaaccaaattaagggccagc	gtgagctgagcagaaggttgga
Sox10 set4 (3HA)	taggtccctcgaagaggttcact	ggacctgaaaggagtgggtagc
Sox10 set5 (Neo)	agcgttggctacccgtgata	aaactcctcctttgccagctc
Sox10 WT	catctcacgccccagttt	tgaggtttcccaccctaccc
Lef1 set1 (671bp)	ctagaagttgggcacccagg	actgtgctgagaacccacag
Lef1 set2 (1.5kb)	gaaaactagcggggttgggt	ttcaatgtgtaggccagggg
Lef1 set3 (2 kb)	tgaactctggtggttggctt	gagacgagatgcaggtggaat
Lef1 set4 (2.2kb)	gatctctgggccagctgaag	ttcaatgtgtaggccagggg

Table S4. Primary antibodies for immunofluorescence staining.

**Antigen**	**Species**	**Dilution**	**Company**	**Cat.No.**

CDX2	Rabbit	1:200	Cell signaling	3977S
OCT4(C-10)	Mouse	1:200	Santa Cruz	Sc-5279
LEF1	Rabbit	1:500	Abcam	ab137872
SOX10	Goat	1:100	Santa Cruz	sc-17342
DsRed	Rabbit	1:100	Abcam	ab62341

Protocol for CRISPR/Cas9 facilitated gene targeting in rat embryonic stem cells

Materials

Advanced DMEM/F12 (Thermo Fisher Scientific 12634010)
B27 (Thermo Fisher Scientific 17504-044)
Beta-mercaptoethanol (Thermo Fisher Scientific 21985023)
CHIR99021 (Stemgent 04-0004)
Fetal bovine serum (Hyclone™ GE Healthcare Life Sciences SH30071.03, or equivalent)
Gelatin (Sigma-Aldrich G1890)
GMEM (Sigma-Aldrich G5154)
γ-irradiated mouse embryonic fibroblasts (iMEFs) E12.5 (prepared in house). We routinely use MEFs prepared from the multidrug resistant DR4 transgenic mice (Tucker et al., 1997)
Leukemia inhibitory factor, recombinant human (prepared in-house, but similar product is supplied by Millipore, LIF1010)
Lipofectamine® 2000 Transfection Reagent (Thermo Fisher Scientific 11668027)
L-glutamine (Sigma-Aldrich G7513)
Matrigel (Corning, 354277)
NEAA (Thermo Fisher Scientific 11140035)
Neurobasal Medium (Thermo Fisher Scientific 21103-049)
N2 supplement (Thermo Fisher Scientific 17502048)
Opti-MEM® I Reduced Serum Medium (Thermo Fisher Scientific 31985062)
Organ culture dishes (Corning 353653)
PD0325901 (Stemgent 04-0006)
Proteinase K (NEB, P8107S)
Rabbit anti-rat antiserum (Sigma-Aldrich R5256)
Rat serum (prepared in house)
Tyrode's solution, acidic (Sigma-Aldrich T1788)
TrypLE™ (Thermo Fisher Scientific 12605028)
Taq DNA polymerase (Qiagen, 201205)
Vacuum filter/storage system (Corning 431098)
Y-27632 (Dihydrochloride) (STEMCELL Technologies, 72304)

Preparation of aN2B27+t2iLY complete medium

Recipe for preparing approximately 1 litre of complete medium:

1. Thaw the N2 and B27 Supplements at room temperature (15 - 25°C) or at 2 - 8°C overnight. Ensure supplements are evenly mixed after thawing.
2. Mix 500ml of advanced DMEM/F12 and 500ml of NeuroBasal.
3. Add 5ml of N2 supplement, 10ml of B27 supplement, 10ml of 200mM L-glutamine solution, 1ml of beta-mercaptoethanol. Mix thoroughly.
4. Filter the medium using a vacuum filter/storage system (Corning™).
5. Keep the aN2B27 medium at 4°C and use within 2 months.
6. Immediately prior to use add small molecules and growth factors to reach the final concentration of 1μM PD0325901, 1μM CHIR99021, 5μM Y-27632 and 10ng/ml human recombinant LIF. The complete medium of aN2B27+t2iLY can be kept at 2 - 8°C for up to one week.

Plating inactivated mouse embryonic fibroblast (iMEF) feeder layers

1. Prepare MEF medium; GMEM with 10% FBS, NEAA, L-glutamine and 0.1mM of beta-mercaptoethanol).
2. Coat plate(s) with 0.1% gelatin and incubate at room temperature for at least 15 minutes.
3. Thaw iMEFs (commercial or prepared in house) and seed onto gelatin-coated plate(s) at a density of approximately 2.5 x 10^4 cells per cm^2. Optimal feeder layer density may vary depending on the cell preparation. Cells should cover the entire surface area of the well 48 hours after plating.
4. Incubate at 37°C overnight to allow the iMEFs to adhere.
5. iMEFs are best used within 2-7 days of plating.

Derivation of rat ES cell lines

The following protocol, modified from (Blair et al., 2012) has been used to derive ES cell lines from Dark Agouti (DA), Wistar and Fischer 344rats. Time-mated rats were purchased from Charles River or matings were set up in house. Females were euthanised on the morning of the 5th day post-coitum (E4.5).

1. Prepare a 96 well plate of iMEF at least one day before derivation and incubate at 37°C (5% O_2, 7% CO_2).
2. On the day of derivation, prepare four organ culture dishes containing aN2B27 in the central well and PBS in the outer well and pre-equilibrate in a humidified incubator at 37°C (5% O_2, 7% CO_2). Prepare and pre-equilibrate one fresh organ culture dish containing aN2B27 + 40% anti-rat serum.
3. Flush embryos from uterus at E4.5 and inspect the embryos. They should be at the mid-blastocyst stage prior to hatching.
4. Prepare a fine drawn pipette using Bunsen burner. Heat the Pasteur pipette in the middle of the thin tube until it glows orange and becomes pliable. Quickly pull the ends of the pipette away from each other to form a straight drawn pipette. Use a forceps to break the tip of the fine drawn pipette under the microscope. The size of the tip should be slightly larger than an E4.5 blastocyst.
5. Place all embryos into a pre-equilibrated organ culture dish containing aN2B27 using a fine drawn pipette. Ensure minimal carry-over of media at each transfer step.
6. Transfer less than 10 embryos at a time into a drop of acidic tyrodes solution in a sterile cell culture dish to remove the zona pellucida. Monitor dissolution of the zona under the dissecting microscope. It should take less than one minute, providing only minimal medium has been carried over.
7. Rinse the embryos in pre-equilibrated aN2B27 and transfer to the dish containing aN2B27 + 40% anti-rat serum.
8. Incubate for 1 hour.
9. Prepare and pre-equilibrate a dish of aN2B27 + 40% rat serum
10. Rinse embryos three times in pre-equilibrated aN2B27.
11. Transfer embryos to dish containing 40% rat serum.
12. Incubate for 20 minutes.
13. Inspect the embryos. The trophoblast layer should have a collapsed and largely disintegrated appearance.
14. Transfer embryos to individual droplets of aN2B27 in a sterile cell culture dish. Remove lysed trophectoderm cells mechanically by trituration using a fine pipette. Lysed trophectoderm can be reserved for genotyping to determine gender.
15. Inspect the immunosurgically isolated ICMs and deposit individually into single wells of a 96 well plate on iMEF in 100 µl of aN2B27 + t2iLY. Add 100 I.U./mL penicillin and 100 (µg/mL) streptomycin. Allow ICM explants to expand for 5-6 days at 37°C (5% O_2, 7% CO_2). Add fresh 100 µl of aN2B27 + t2iLY (without antibiotics) to each well at day 3.

16. At day 5-6, wells containing outgrowths are individually passaged 1:1 into new wells of a MEF coated 96 well plate. For passaging, aspirate the medium and add 50 μl/well of TrypLE™ Express into each well and incubate the plate at 37°C for 3 minutes. Add 150 μl /well of wash buffer (DMEM/F12 + 2% serum) and pipette up and down to stop trypsinisation and dissociate the outgrowth into single cells or doublets. Transfer cell suspension into a 96-well V-bottom plate and spin down using a plate centrifuge. Resuspend each cell pellet in 200 μl of culture medium (aN2B27+t2iLY) and seed into the new plate.
17. Colonies with domed morphology should become visible within 3 days after passaging and can subsequently be passaged every 2-3 days at ratio of 1:2 to 1:4. Gradually expand lines to progressively bigger wells. At early passages cells grow better plated into small wells.
18. Expanding cultures may be considered established ES cell lines from passage 4 onwards. Note that each line is derived from a single ICM outgrowth.
19. Freeze using medium containing 10% DMSO, 10% serum and 80% rat ES cell culture medium.

Passaging established rat ES cells
Rat ES cells are normally passaged every 48 hours with no medium change is required in between. Always passage before confluence and never allow cultures to over-grow, as cell viability will drop significantly and karyotypic changes may ensue. When assessing confluency, always check the edges of the well since these areas usually have higher cell density. The colonies are ready to be passaged when they reach 50 - 70 µm in diameter. A healthy colony has a smooth round surface. If colonies develop a roughened surface (see arrowed colonies in Figure) cells will not recover well after passaging. Therefore, if raspberry-like colonies appear in the culture, passage immediately. 12-well plates are recommended for routine rat ES cell maintenance because minimal circulation of medium reduces colony detachment.

Figure. Healthy (left) and unhealthy (right) rat ES cell cultures (scale bar: 100µm)

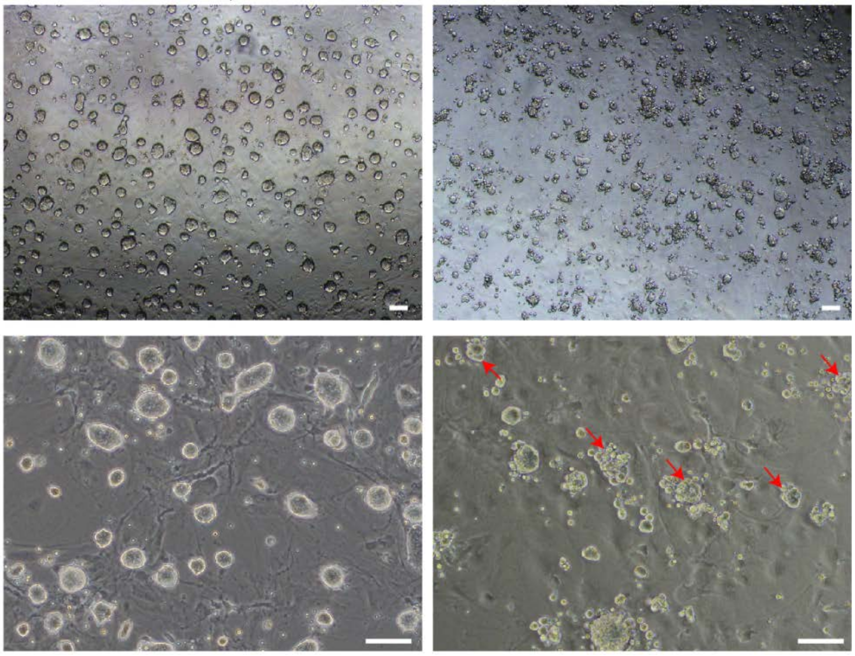

12-well plates are recommended for routine rat ES cell maintenance because minimal circulation of medium reduces colony detachment.

1. Prepare iMEF-coated plates at least one day prior to plating ES cells.

2. Warm aN2B27+t2iLY and wash buffer to room temperature before use.
3. Inspect rat ES cell culture before passaging. If significant detachment of colonies from the feeder layer is observed, collect all the culture medium from the well and spin at 300 x *g* for 3 minutes to recover those detached colonies. Resuspend them in TrypLE.
4. Otherwise, aspirate culture medium from wells containing rat ES cells. Try to remove as much residual medium as carefully as possible. It is not recommended to wash the culture with PBS since rat ES cells are loosely attached and can be lost during the wash step.
5. Add TrypLE and incubate at 37°C for 3 minutes. Recovered floating colonies can be added back to the wells they were harvested from. Allow them to be incubated at 37°C for an extra 1.5 minutes. Pipette up and down to dissociate the colonies. Try to avoid bubbles during pipetting.
6. Transfer to a 15mL conical tube. Add 4 volumes of wash buffer to neutralise TrypLE and centrifuge at 300 x *g* for 3 minutes.
7. Aspirate the wash buffer. Resuspend the cell pellet in 1mL of aN2B27+t2iLY medium.
8. Aspirate MEF medium from iMEF-coated plate. Try to remove as much MEF medium as possible.
9. Dispense rat ES cell suspension into the wells according to desired passage ratio. Top up aN2B27+t2iLY medium to 0.5mL per cm^2 (approximately 2mL per well of a 12 well plate. A healthy rat ES cell culture will typically be passaged every 2 days at a split ratio of 1:4.
10. Distribute rat ES cells evenly across the well by moving the plate in several short, back-and-forth and side-to-side motions. Incubate at 37°C (5% O2, 7% CO2).
11. Visually assess cultures daily to monitor growth until the next passage. Avoid shaking the plate as colonies may detach.

Lipofection and selection

Rat ES cells can be transfected using different methods. Following is a protocol using Lipofectamine® 2000. Note that less DNA is used for CRISPR experiments than standard transgene transfection in order to minimise undesired random integration. Plan your CRISPR constructs; design sgRNAs using tools such as http://crispr.mit.edu:8079/. Choose an expression vector to clone sgRNA-encoding plasmids. For example, we used U6-BsaI-sgRNA backbone (kindly provided by S. Gerety, Sanger Institute, Cambridge, UK). For Cas9-encoding plasmids, we chose hCas9_D10A Cas9 (a gift from George Church (Addgene plasmid # 41816)), which expresses Cas9 nickase.

1. Prepare a 12-well plate of rat ES cells (approximately 1 x 10^6 cells in total) for transfection.
2. Plate a full 6-well plate of iMEFs and four 10-cm dishes of DR4 (resistant to neomycin, hygromycin, puromycin and 6-thioguanine) iMEFs at least 1 day prior to transfection.
3. Prepare Matrigel coating on iMEF dishes. Matrigel provides better attachment of rat ES cells during selection. Add 100 μL of Corning Matrigel hESC-Qualified Matrix to 24mL of cold MEF medium and mix well. Aspirate the MEF medium from the dishes and add 6 mL of Matrigel containing medium to each dish. Incubate at 37°C for at least 8 hours before use.
4. For transfection prepare six aliquots of 200 μL Opti-MEM® I Reduced Serum Medium in Eppendorf tubes.
5. Add 12 μL of Lipofectamine® 2000 Reagent into each tube. Tap tube to mix well.
6. Prepare a mixture of 1.2 μg of sgRNA plasmid, 1.2μg of Cas9 plasmid, 1.2μg of targeting vector in 1.3 mL of Opti-MEM® I Reduced Serum Medium in a new Eppendorf tube. Pipette to mix well.

7. Dispense 200 μL of plasmid mixture into each tube of Lipofectamine® 2000 Reagent mixture. Tap tube to mix well. Incubate at room temperature for 20 minutes.
8. Dissociate rat ES cells using TrypLE as for routine passaging and transfer cell suspension 1:1 onto a new 6-well plate of iMEF.
9. Immediately add 400 μL of DNA-lipid complex dropwise to each well of the plate. Move the plate in several short, back-and-forth and side-to-side motions then return to incubator (5% O2, 7% CO2). Incubate for 8-10 hours before passaging. Please note that incubation in lipofection media for longer than 10 hours will result in decreased cell viability.
10. Passage transfected rat ES cells onto four Matrigel-coated 10cm DR4 iMEF dishes.
11. Start selection 24 hours after passaging using lowest drug concentration that completely eliminates wildtype cells.
12. Perform daily medium change for the first 4 days and subsequently change the medium every other day until the colonies are large enough for picking.

Colony picking and expansion

It usually takes 7-8 days from the start of the selection for colonies to reach 100 to 150μm in diameter, which is optimal for picking. Colonies smaller than this may be difficult to pick and may have few viable cells after picking. Larger colonies may accumulate dead cells in the middle of the colony and have higher level of spontaneous differentiation after passaging. Please note that colonies may reach size for picking on different days.

1. Plate down DR4 iMEFs on several flat-bottom 96 well plates at least 48 hours before colony picking.
2. Ideally use a fine drawn glass pipette for picking colonies. Otherwise, use a P200 pipette.
3. Prepare several U or V-bottom 96 well plates with 30 μL/well of TrypLE.
4. Pick colonies under a microscope and deposit individually into single wells of prepared 96 well plates with TrypLE. Try to pick as many colonies as possible.
5. Dispense 200 μL of wash buffer into each well and use a multi-channel pipette to pipette up and down to dissociate the colonies.
6. Spin down at 1200rpm for 3 minutes using a plate centrifuge.
7. Resuspend each cell pellet in 200 μl of complete medium supplemented with selection drug (e.g.neomycin at 300 μg/mL) and antibiotics and transfer onto DR4 iMEFs coated 96 well plates.
8. Incubate for 2-3 days. Inspect each well on day 2 and day 3. Note that different wells may require passage on different days depending on cell number and colony size.
9. Passage each well when ready into two wells on separate DR4 iMEF coated 96-well plates. Maintain selection but withdraw antibiotics. The duplicate 96-well plate is used for genotyping.
10. Once correct genotype is confirmed, gradually expand the clones from 96 well plates to progressively larger wells.

Primary genotyping

1. When the plate for genotyping becomes confluent, aspirate medium from the wells. Add 50μL of ES cell lysis buffer (100mM pH 8.5 Tris-Cl, 5mM EDTA, 0.2% SDS, 200mM NaCl, 100μg/ml Proteinase K) into each well and incubate at 55°C for at least 3 hours.
2. Add 5μL of 3M sodium acetate (pH5.2) and 100μL of ethanol into each well. Let genomic DNA precipitate at room temperature for 1-2 hours. Do not mix or shake the plate. Precipitated genomic DNA will stick to the wall of the wells. Invert the plate to decant the solution. Gently wash the well with 70% ethanol. Invert the plate to decant the ethanol and allow the plate to air dry.

3. Dissolve genomic DNA in the wells in 100µL of TE buffer. Proceed to genomic PCR using Qiagen Taq polymerase. Avoid using high efficiency DNA polymerases for this purpose as they are likely to amplify products from non-specific binding of primers to the genome.
4. When designing genotyping primer pairs for homologous recombination, one primer must be specific to the introduced sequence while the other primer corresponds to genomic sequence flanking the homology arm. When designing genotyping primer pairs for non-homologous end joining (NHEJ), the two primers of each primer pair should flank the genomic region of the CRISPR/Cas recognition sequence. It may be necessary to design primers at varying distances from the target site to define deletions resulting from NHEJ.
5. Identify correctly targeted clones by agarose gel electrophoresis. Confirm by sequencing the PCR product.

References

Blair, K., Leitch, H.G., Mansfield, W., Dumeau, C.E., Humphreys, P., and Smith, A.G. (2012). Culture parameters for stable expansion, genetic modification and germline transmission of rat pluripotent stem cells. Biology Open *1*, 58-65.

Tucker, K.L., Wang, Y., Dausman, J., and Jaenisch, R. (1997). A transgenic mouse strain expressing four drug-selectable marker genes. Nucleic Acids Research *25*, 3745-3746.
