# Supplementary material for: Gene Editing in Rat Embryonic Stem Cells to Produce In Vitro Models and In Vivo Reporters
Source: Stem Cell Reports. 2017 Oct 10;9(4):1262–74. doi: 10.1016/j.stemcr.2017.09.005 (PMC5639479; doi:10.1016/j.stemcr.2017.09.005)
Supplement: Document S2. Article plus Supplemental Information [file mmc2.pdf]

Stem Cell Reports
Article

OPEN ACCESS

Gene Editing in Rat Embryonic Stem Cells to Produce *In Vitro* Models and *In Vivo* Reporters

Yaoyao Chen,[1] Sonia Spitzer,[1,2] Sylvia Agathou,[1,2] Ragnhildur Thora Karadottir,[1,2] and Austin Smith[1,3,*]
[1]Wellcome Trust-Medical Research Council Cambridge Stem Cell Institute, University of Cambridge, Tennis Court Road, Cambridge CB2 1QR, UK
[2]Department of Veterinary Medicine, University of Cambridge, Madingley Road, Cambridge CB3 0ES, UK
[3]Department of Biochemistry, University of Cambridge, Tennis Court Road, Cambridge CB2 1GA, UK
*Correspondence: austin.smith@cscr.cam.ac.uk
http://dx.doi.org/10.1016/j.stemcr.2017.09.005

SUMMARY

Rat embryonic stem cells (ESCs) offer the potential for sophisticated genome engineering in this valuable biomedical model species. However, germline transmission has been rare following conventional homologous recombination and clonal selection. Here, we used the CRISPR/Cas9 system to target genomic mutations and insertions. We first evaluated utility for directed mutagenesis and recovered clones with biallelic deletions in *Lef1*. Mutant cells exhibited reduced sensitivity to glycogen synthase kinase 3 inhibition during self-renewal. We then generated a non-disruptive knockin of *dsRed* at the *Sox10* locus. Two clones produced germline chimeras. Comparative expression of dsRed and SOX10 validated the fidelity of the reporter. To illustrate utility, live imaging of dsRed in neonatal brain slices was employed to visualize oligodendrocyte lineage cells for patch-clamp recording. Overall, these results show that CRISPR/Cas9 gene editing technology in germline-competent rat ESCs is enabling for *in vitro* studies and for generating genetically modified rats.

INTRODUCTION

The rat *Rattus* is a valuable and widely used model organism for studying cognition and behavior, physiology, toxicology, and various pathologies, such as metabolic and neurodegenerative diseases (Iannaccone and Jacob, 2009). Although the rat was the first mammalian species to be domesticated for biomedical research (Jacob et al., 2010), it has been outpaced in recent years by the mouse, in part because of limitations in directed manipulation of the rat genome. In mice, genome engineering is mostly performed via embryonic stem cells (ESCs), and the ease of carrying out such work has been key to their widespread use as an animal model (Capecchi, 2005). Following the definition of culture requirements for mouse ESCs (Ying et al., 2008), rat ESCs have been derived from different rat strains using similar conditions (Buehr et al., 2008; Hirabayashi et al., 2010a; Li et al., 2008). However, rat ESCs are less robust than their mouse counterparts and demand expert handling to maintain robust growth and capacity for germline transmission (Blair et al., 2011), especially after clonal selection required for gene targeting (Hirabayashi et al., 2010b, 2013, 2014; Meek et al., 2010; Men et al., 2012; Men and Bryda, 2013; Tong et al., 2010). These technical difficulties have hindered the widespread adoption of rat ESC transgenesis.

Meanwhile, the development of the CRISPR/Cas9 system (Cho et al., 2013; Cong et al., 2013; Hwang et al., 2013; Ma et al., 2014; Mali et al., 2013; Shen et al., 2013; Wang et al., 2013; Yang et al., 2013) has enabled rat genome editing via direct injection of one-cell embryos (Kim and Kim, 2014; Li et al., 2013a, 2013b; Ma et al., 2014; Shao et al., 2014). The injected endonuclease is targeted to a specific DNA sequence by guide RNAs (gRNAs) and introduces double-strand breaks, which can be repaired by non-homologous end-joining (NHEJ) (Garneau et al., 2010; Lieber, 2010; Marraffini and Sontheimer, 2010). Error-prone NHEJ generally introduces small indels at the cleavage site to generate mutation in one or both alleles of the target sequence. Several knockout rats have been generated using this method (Li et al., 2013a, 2013b). More recently, insertion of large DNA fragments at target loci has been achieved using single-stranded oligodeoxynucleotides (ssODNs) together with CRISPR/Cas9 (Chen et al., 2011; Storici et al., 2006; Yoshimi et al., 2014, 2016). However, targeting efficiency varies unpredictably between different loci and according to the size of the insert. Moreover, both methods are inefficient and require injections of large numbers of embryos with associated maintenance of substantial numbers of animals. Furthermore, first-generation animals are generally mosaic, necessitating additional breeding and genotyping. Therefore, this approach does not provide the most efficient use of animals consistent with the 3R principles of reduction, refinement, and replacement. CRISPR/Cas9-mediated gene editing has also been applied in spermatogonial stem cells to create knockout rats (Chapman et al., 2015). Germline genome editing can avoid the production of mosaic mutant progeny (Brinster and Avarbock, 1994). However, homologous recombination has yet to be demonstrated, which limits applications.

Here, we tested whether CRISPR/Cas9 technology can be applied in rat ESCs both for *in vitro* studies and for generation of rats with targeted genomic insertions.

 © 2017 The Authors.
This is an open access article under the CC BY license (http://creativecommons.org/licenses/by/4.0/).

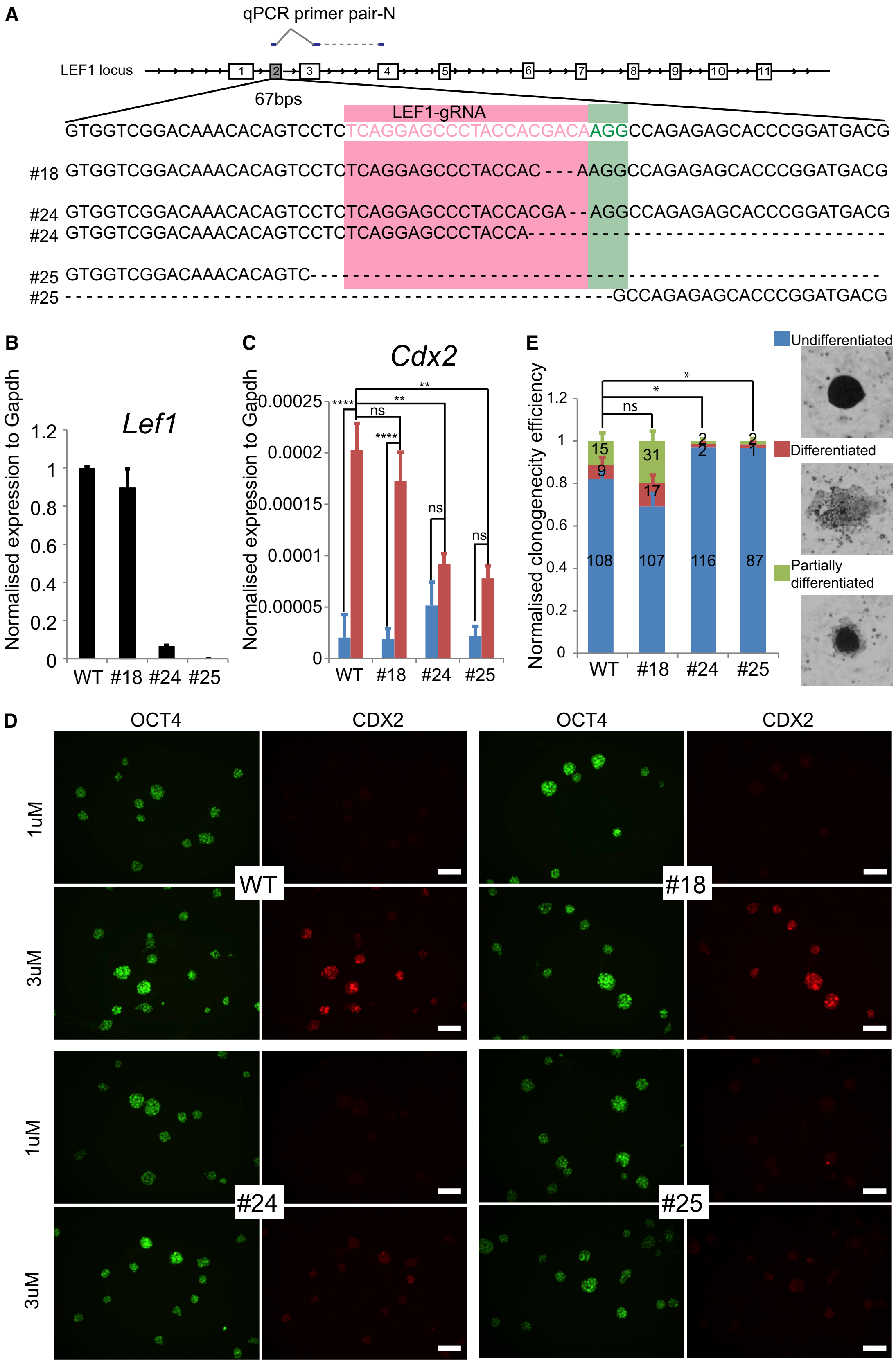

(legend on next page)

RESULTS

Rat Embryonic Stem Cell Derivation and Culture

The culture conditions for rat ESCs were previously adjusted to reduce spontaneous differentiation by lowering the concentration of the glycogen synthase kinase-3 (GSK3) inhibitor CHIR99021 (CH) (Chen et al., 2013; Meek et al., 2013). However, even under these culture conditions, termed t2iL (see Experimental Procedures), rat ESCs still exhibit unreliable attachment to feeders, inconsistent growth rate and viability during routine passaging, sporadic differentiation, and a tendency to become tetraploid. These issues pose particular concern during the stringent clonal selection and expansion required for gene targeting. Therefore, we assessed several parameters during derivation of new ESC lines from Dark Agouti rats in t2iL. Conditions tested were: addition of the PKC inhibitor Gö6983 (Rajendran et al., 2013); addition of vitamin C (250 μM) (Esteban et al., 2010); use of Rho-associated kinase inhibitor Y-27632 (Watanabe et al., 2007); substitution of DMEM/F12 with lipid-rich advanced DMEM/F12; reduced oxygen atmosphere. We found that establishment of cell lines was most reliable using advanced DMEM/F12 in the base N2B27 formulation (Ying et al., 2003) together with t2iL, and with addition of Y-27632 in 5% O_2. We selected one of the newly derived female ESC lines, DAC27, for use in subsequent experiments.

We first re-tested the effect of the empirical culture modifications on colony formation from single DAC27 cells. Advanced DMEM/F12 and reduced oxygen gave modest but additive improvements (Figure S1). Addition of Rho-associated kinase inhibitor Y-27632 (Watanabe et al., 2007) had a more substantial effect. The combination of aDMEM/F12, 5% O_2 and Y-27632 gave a colony-forming efficiency of around 80% and moreover made routine passaging more consistent. We therefore incorporated all three modifications into the culture system for targeted genome modification.

Targeted Mutation of *Lef1*

Expression of the canonical Wnt signaling effector *Lef1* has been proposed to underlie the hypersensitivity of rat ESCs to the GSK3 inhibitor CH (Chen et al., 2013). We therefore chose the *Lef1* gene to test the applicability of CRISPR/Cas9 for targeted gene mutation in rat ESCs. A gRNA was designed using the CRISPR Design tool (http://crispr.mit.edu/) to target the second exon of *Lef1* (Figure 1A).

For *Lef1* targeting, 1×10^6 rat ESCs were transfected using Lipofectamine 2000 with 1.2 μg of expression plasmid containing gRNA and Cas9-2A-GFP. Eight hours post transfection, cells were replated onto new feeders in fresh medium. Twenty-four hours after replating, GFP-positive cells were sorted by flow cytometry into 10 cm culture dishes at a density of 10,000 cells per dish. Fifteen milliliters of medium was added into each dish, and no medium change was required thereafter. Five days later, individual colonies were picked, plated into duplicate 96-wells, and expanded briefly before genotyping one of the duplicates.

Genomic PCR followed by gel electrophoresis indicated that 5 of 38 (13%) expanded cultures had an overt deletion in one or both alleles of *Lef1* (Figure S2A). We selected three clones with distinct gPCR products: cl18, no overt size change; cl24, one smaller band; cl25, no wild-type band. We subcloned 24 and 25 and repeated the gPCR screen to eliminate the possibility of mixed colonies from the primary plating. We then sequenced the genomic region spanning *Lef1* exon2. Clone 18 had an in-frame deletion of 3 bp, with no wild-type sequence. Clone 24 had a 2 bp frameshift mutation in one allele and a deletion of 124 bp in the other allele. Clone 25 had deletions of 173 and 506 bp (Figure 1A).

To assess whether *Lef1* expression was indeed disrupted in these three clones, we designed primers flanking the gRNA recognition site and performed RT-qPCR analysis (Figure 1B). Clone 18 yielded a PCR product in similar amount to parental cells. Consistent with sequencing results, clone 25 yielded no detectable product. Analysis of clone 24, on the other hand, indicated a residual level of transcript. This could be due to incomplete nonsense mediated mRNA decay of the frameshifted transcript. We examined LEF1 protein expression by immunocytochemistry using a monoclonal antibody that detects an epitope downstream of the deleted region encoded by exon 2.

Figure 1. Generation and Characterization of *Lef1* Knockout rESCs

(A) Design for CRISPR/Cas9-targeted mutation of *Lef1* exon2 and sequence of targeted clones.

(B) Expression of *Lef1* transcript in parental and *Lef1* mutant rat ESCs assayed by RT-qPCR. Error bars represent the SD from three technical replicates.

(C) Expression level of *Cdx2* transcript in response to CH at 1 μM or 3 μM. Error bars represent the SD of three independent experiments. Expression was normalized to *Gapdh*. ANOVA statistical analysis indicates the effect of CH significant ($p < 0.0001$) in wild-type (WT) and clone 18 but not in LEF1 mutant clones (clone 24, $p = 0.4711$; clone 25, $p = 0.1567$; ns, not significant; **$p < 0.01$, ****$p < 0.0001$).

(D) Fluorescent immunostaining of CDX2 and OCT4. Scale bars represent 100 μm.

(E) Colony-forming assay in the presence of 3 μM CH. Undifferentiated, partially differentiated, and differentiated colonies were calculated relative to the total number of colonies counted for each line. Error bars represent SD from three biological replicates. *$p < 0.001$ (ANOVA).

Strong staining was observed in parental and clone 18 cells, while clone 24 and clone 25 cells were unstained (Figure S2B). Collectively, these data indicate that clones 24 and 25 are null mutants lacking LEF1.

We examined the phenotypic consequence of loss of LEF1. In standard 2iLIF medium containing 3 μM CH (Ying et al., 2008), expression of Wnt targets related to differentiation, such as CDX2, is appreciable in rat ESCs (Chen et al., 2013; Meek et al., 2013). To investigate whether inactivating *Lef1* could alleviate the hypersensitivity of rat ESCs to GSK3 inhibition, we first measured the induction of *Cdx2* by RT-qPCR. In parental cells, the expression of *Cdx2* increased more than 6-fold when CH concentration was raised from 1 μM to 3 μM. Clone 18 cells showed a similar response to CH, suggesting that loss of a single amino acid has a minor effect on LEF1 function. In contrast, the *Cdx2* response to CH was reduced in clones 24 and 25 (Figure 1C). Expression of CDX2 protein was also markedly attenuated in these two *Lef1* mutant clones, as shown by immunofluorescence staining (Figure 1D).

To assess whether loss of *Lef1* had an impact on the self-renewal of rat ESCs, we performed colony-forming assays in the presence of 3 μM CH. Colonies were stained for alkaline phosphatase and scored for level of differentiation, categorized as undifferentiated, partially differentiated, or differentiated. A representative image of each category is shown in Figure 1E. As previously reported (Chen et al., 2013), differentiation was overt in around 20% of parental rat ESCs cultured in 3 μM CH. This was also apparent in clone 18. In contrast, in clone 24 and clone 25 mutants, fewer than 5% of colonies contained differentiated cells (Figure 1E). We also observed that clones 24 and 25 could be propagated readily in standard 2iL with no evident detriment compared with t2iL, in contrast to parental or clone 18 cells. These results are consistent with LEF1 mediating differentiation sensitivity of rat ESCs to GSK3β inhibition.

Generation of a Non-disruptive *Sox10* Knockin Reporter

Based on the proof of principle of genome editing in rat ESCs, we sought to generate a targeted knockin modification via CRISPR/Cas9-facilitated homologous recombination. We chose the *Sox10* gene in order to create a reporter rat of value to the developmental biology and neuroscience communities. *Sox10* is a member of the Sry-related HMG box (Sox) family of transcription factors. It is expressed throughout the developing neural crest (Kelsh, 2006) and in all oligodendroglial lineage cells (Stolt et al., 2002). One particular attraction of a *Sox10* reporter is as a tool for visualizing and isolating precursor and mature oligodendrocytes from postnatal animals. Indeed several transgenic mouse lines have been created using the *Sox10* promoter (Kessaris et al., 2006; Rinholm et al., 2011; Shibata et al., 2010; Simon et al., 2012). However, rats in which oligodendrocyte lineage cells are specifically labeled would be a valuable resource due to the relative ease of surgical procedures (Iannaccone and Jacob, 2009) and superiority of demyelinating lesions models in the rat (Woodruff and Franklin, 1999), combined with their greater suitability for learning and cognition assays (Iannaccone and Jacob, 2009).

In common with several other *Sox* gene family members, *Sox10* is haploinsufficient (Britsch et al., 2001; Paratore et al., 2002). It is therefore essential to avoid disruption of endogenous SOX10 expression in any knockin reporter. We designed a construct to insert an internal ribosome entry site (IRES) coupled to a red fluorescent protein (HisDsRed) coding sequence into the 3′ UTR, leaving the *Sox10* gene structure intact (Figure 2A). dsRed is fused to a histidinol resistance enzyme, allowing the potential option for drug selection of *Sox10*-expressing cells if required. The insertion site was selected 5 bp downstream of the stop codon. Fragments of approximately 1.2 kb of genomic sequence were amplified by genomic PCR to generate 5′ and 3′ homology arms. *Sox10* is not expressed in ESCs; therefore, positive selection was provided by a PGK-Neo cassette flanked by *Loxp* sites.

We designed two gRNAs with recognition sites close to the designated insertion site in the 3′ UTR. We introduced the gRNAs together with the *Sox10-IRES-HisDsRed* targeting vector and *Cas9* nickase plasmid into 1×10^6 DAC27 rat ESCs via lipofection. Use of Cas9 nickase is reported to increase the ratio of homology-directed repair to NHEJ and reduce off-target genome disruption (Cong et al., 2013; Mali et al., 2013; Rong et al., 2014). Transfected cells were replated after 8 hr into 4 × 10 cm dishes on feeders overlaid with Matrigel. After 24 hr, G418 (300 μg/mL) selection was applied. Colonies were picked after 7 days and expanded without further selection in duplicate for genotyping.

Two of 52 picked colonies, 3B and 6G, yielded a band of the expected size (~1.4 kb) for homologous recombination detected by genomic PCR using a primer pair flanking the 5′ homology arm (Figure 2B). These two clones were validated further using primers to amplify the reporter region and the 3′ homology arm (Figure S3). To check whether CRISPR/Cas9 editing had created undesired mutations close to the gRNA recognition sites, we sequenced these genomic regions. No mutations were detected in either clone (Figure 2C).

We assessed the chromosome complement of the two clones by metaphase analysis. Clone 3B was comprised of hyperdiploid and tetraploid cells (Figure 2D) and was discarded. Clone 6G had a proportion of tetraploid cells, but 13 out of 28 spreads examined (46%) had a euploid count of 42 chromosomes (Figure 2D). This clone was therefore chosen to proceed to the next step. Cells were

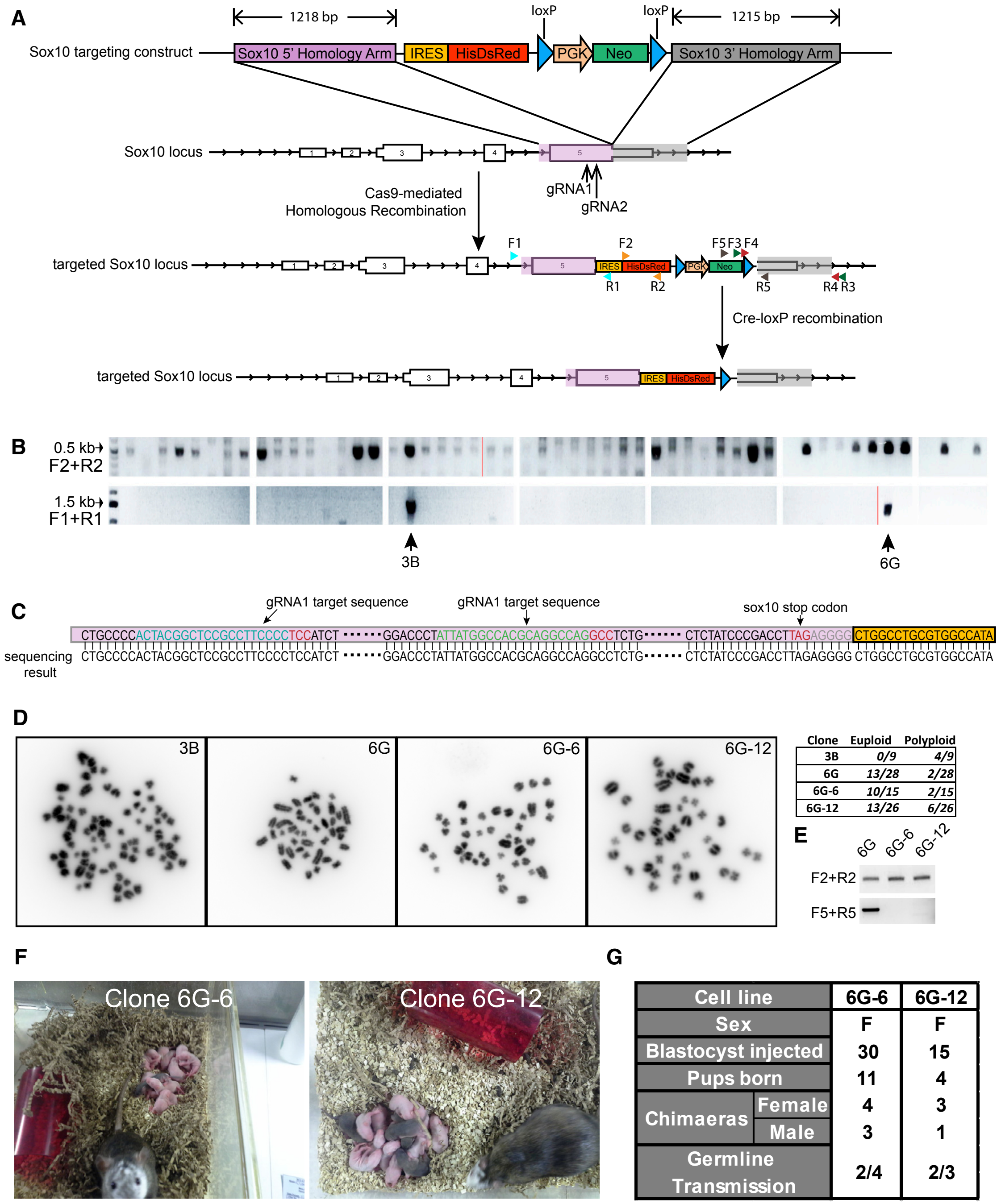

Figure 2. Generation of Sox10-dsRed Reporter Transgenic Rat

(A) Design of *Sox10* targeting.

(B) Screen for targeting by genomic PCR.

(C) Genomic sequence around gRNA recognition sites in clone 6G cells.

(D) Representative images and chromosome counts of metaphase spreads in Sox10 targeted clones.

(E) Genomic PCR assay for excision of PGK-neo^R selection cassette

(F) Chimeras and germline F1 pups following injection of Dark Agouti ESCs 6G-6 and 6G-12 into SD (albino) blastocysts.

(G) Summary of chimeras and test breeding.

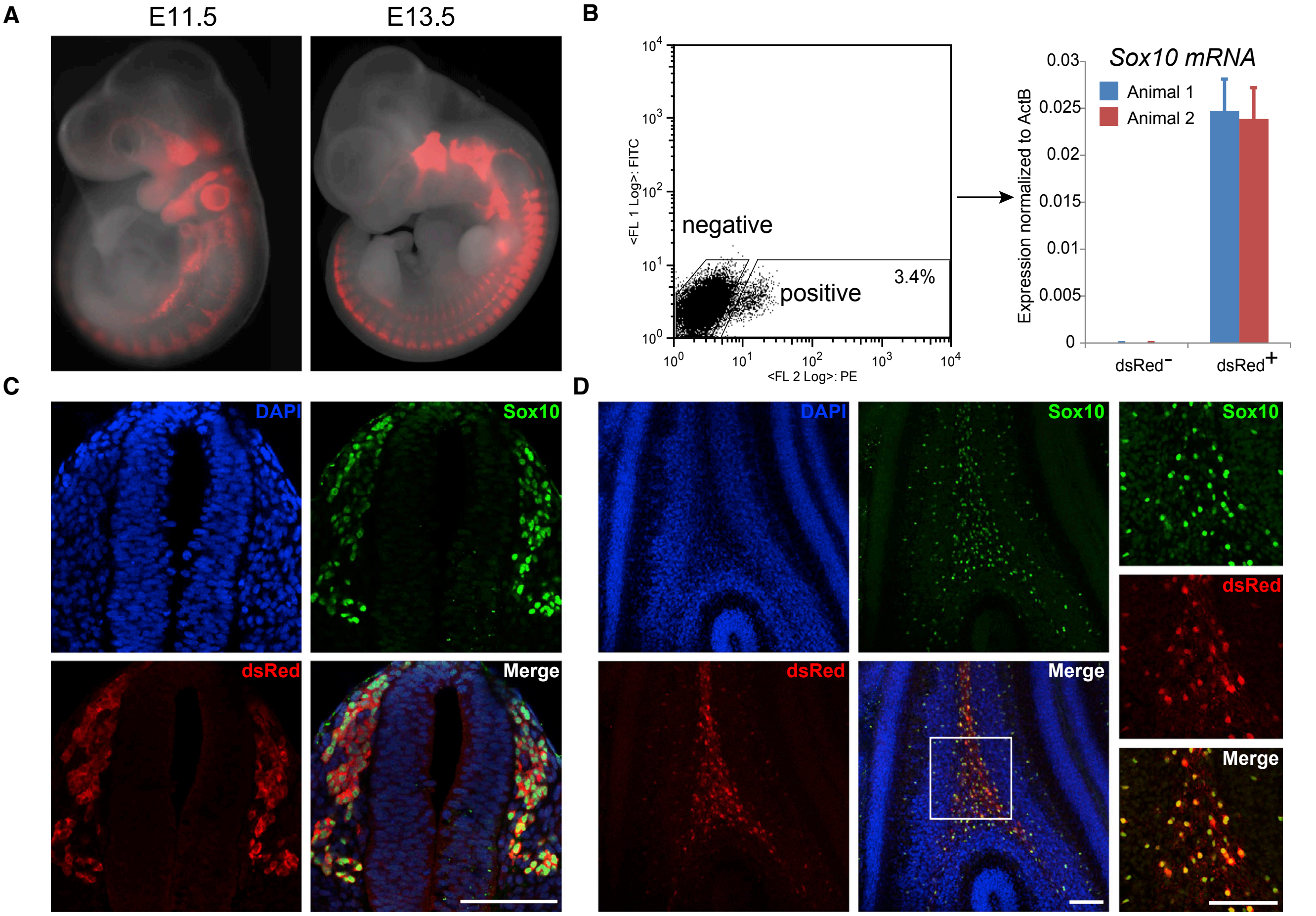

Figure 3. Characterization of Sox10-dsRed Reporter Transgenic Rat

(A) Fluorescent images of E11.5 and E13.5 embryos.

(B) Live sorting of dsRed$^-$ and dsRed$^+$ cells from E11.5 embryos followed by RT-qPCR for *Sox10* transcript. Error bars represent the SD from three technical replicates.

(C) Immunostaining for dsRed and SOX10 on 100 μm cross-sections of the spinal cord region at E13.5. Scale bars represent 100 μm.

(D) Immunostaining of 100 μm cross-sections of P7 newborn rat cerebellum. The white boxed area is shown in higher magnification in the right panel. Scale bars represent 100 μm.

transfected with a Cre recombinase expression plasmid and subsequently plated at low density (10,000 cells per 10 cm dish) for sub-cloning. Individual colonies were picked and split into duplicate wells of a 96-well plate and cultured with or without G418. Loss of resistance to the antibiotic indicated excision of the PGK-Neo cassette.

Chromosome counts were again checked by metaphase analysis. Two of 12 clones, clone 6G-6 and clone 6G-12, contained at least 50% euploid cells (Figure 2D). Genomic PCR confirmed the absence of PGKneo^R in both clones (Figure 2E). They were expanded briefly before injection into blastocysts of the albino SD strain. Coat color chimeras were obtained in both cases (Figure 2F). Female chimeras were test mated to SD males and from each clone, two animals proved to be germline competent in the first litter (Figures 2F and 2G). These data demonstrate that rat ESCs maintained using aN2B27-t2iLY in 5%O_2 can maintain full competence after two rounds of genetic engineering and clonal selection.

Sox10 Reporter Characterization

Germline offspring from both knockin clones were bred with SD animals to establish transgenic lines. Heterozygous outcross matings were employed to characterize reporter expression. We evaluated the pattern of dsRed fluorescence at two developmental stages. At embryonic day 11.5 (E11.5), dsRed signal was readily detected in neural crest cell derivatives and the otic placode (Figure 3A), consistent with the pattern of SOX10 expression during embryonic development (Breuskin et al., 2009, 2010). At E13.5,

fluorescence signals were prominent in dorsal root ganglia (DRG) and trigeminal ganglia, as expected.

We dissociated E11.5 embryos into single cells and sorted dsRed-positive and -negative populations by flow cytometry. Approximately 3.5% live cells were positive for dsRed fluorescence. The positive and negative populations were analyzed for *Sox10* mRNA by RT-qPCR (Figure 3B). *Sox10* transcript was only detected in the dsRed-positive population, indicating faithful reporting of endogenous *Sox10* transcripts. We carried out double immunofluorescent staining for SOX10 and dsRed proteins on sections from E13.5 embryos and P7 newborn rat brain cerebellum. As shown in Figures 3C and 3D, respectively, SOX10 antibody stained nuclei in the DRG region of E13.5 embryos and the cerebellum of P7 neonates. dsRed was detected in the cytoplasm of the same subset of cells. Notably, we did not observe expression of dsRed without co-expression of SOX10.

Following outcrossing of F1 animals, we set up intercross matings to acquire homozygotes. Homozygous animals were obtained from both clones. We euthanized a homozygote at 14 weeks and prepared brain sections. dsRed-positive cells were evenly distributed across the cortex, corpus callosum, and sub-cortical regions (Figure S4A). Immunostaining for myelin basic protein indicated a normal pattern of myelin deposition in white and gray matter (Figures S4B–S4D). Co-expression of dsRed was detected in Olig2-positive cells and in NG2-positive oligodendrocyte progenitors (Figures S4E–S4H).

Mice and humans heterozygous for *Sox10* loss of function mutations display overt phenotypes: abnormal pigmentation and megacolon in mice; Hirschsprung disease in humans (Britsch et al., 2001; Paratore et al., 2002). Such haploinsufficiency implies that SOX10 protein dosage is critical. In contrast, we have observed no abnormalities in multiple heterozygous and homozygous animals. We surmise that *Sox10::dsRed* knockin rats express functional SOX10 at physiologically sufficient levels. We cryopreserved embryos derived from clone 6G-12 and have deposited live rats with the Rat Resource & Research Center.

Whole-Cell Patch-Clamp Recording from dsRed-Positive Oligodendrocyte Lineage Cells

We examined whether this reporter rat can facilitate the study of oligodendroglial cells. First, we checked whether a dsRed signal can be detected in living cells in postnatal rat brain. In freshly prepared coronal brain slices, we identified dsRed$^+$ cells by fluorescence microscopy in both cortex (gray matter) and corpus callosum (white matter). Positive cells displayed morphology of oligodendrocyte lineage cells (Figure 4A). All stages of oligodendroglia are expected to be labeled by *Sox10*:*dsRed*. To confirm this, fluorescent cells were selected for whole-cell voltage-clamp recordings. Cells of different lineage stages were identified based on morphology assessed through live-cell imaging. Once cells were patched in whole-cell mode, lineage stage was further evaluated by morphology through additional lucifer yellow dye labeling. The current response evoked by a 10 mV pulse (150 ms duration) was used to determine the decay constant and input resistance, and voltage-current membrane properties were used to analyze voltage-gated sodium and potassium currents. Cartoons and live images of cortical oligodendrocyte progenitor cells are shown in Figure 4B. Representative recordings of early oligodendrocyte progenitors, mature oligodendrocyte progenitors, and fully differentiated oligodendrocyte are shown in Figures 4C–4E. The electrophysiological properties are consistent with the morphological assessments of maturation stage.

Oligodendrocyte lineage cells express glutamate receptors including kainate receptor (Verkhratsky and Steinhauser, 2000) and respond to kainate stimulation (Figure 4F). Therefore, we also measured the kainate response in dsRed$^+$ cells. More than 80% showed a response, further indicating that *Sox10*:*dsRed* identifies functional oligodendrocytes in the brain.

DISCUSSION

Here, we have documented the application of CRISPR/Cas9-mediated genome editing in rat ESCs and demonstrated the utility for generation of *in vitro* and *in vivo* models. Incremental refinements of rat ESC culture conditions conferred more consistent growth and clonogenicity, facilitating recovery of clones after genetic manipulation. In addition, we took two further measures to maximize the probability of germline transmission: first, we used rat ESCs at low passages; second, we selected diploid clones after each round of clonal selection. Combined with use of the CRISPR/Cas9 system, these refinements increase the practicality of using rat ESCs for gene targeting. Although the incidence of *Sox10* knockin was only 4% of stable transfectants, the construct used lacked any negative selection cassette, commonly included to enrich for homologous recombinants. Ease of vector preparation due to shorter homology arms is an advantage of using Cas9 compared with conventional homologous recombination. More importantly, both of the targeted sub-clones selected for blastocyst injection gave high-contribution chimerism and germline transmission.

The creation of *Lef1* mutant rat ESCs allowed examination of the significance of *Lef1* downstream of GSK3 inhibition. This analysis provided further evidence that *Lef1* contributes to destabilization of self-renewal via induction

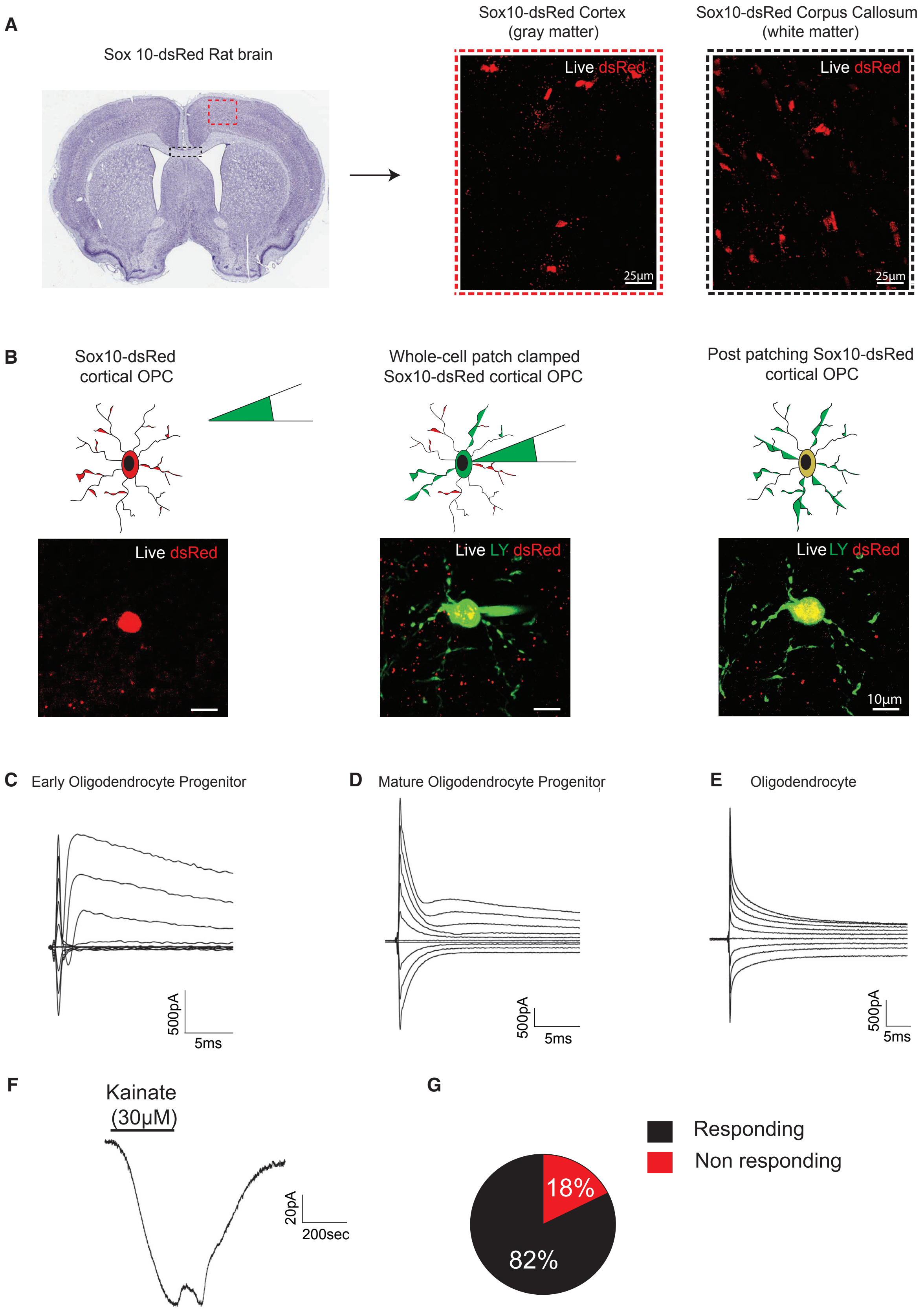

(legend on next page)

of lineage specification genes downstream (Chen et al., 2013; Meek et al., 2013). These results may explain why the optimal concentration of GSK3 inhibitor CH is only 1 μM compared with 3 μM for mouse ESCs. Interestingly, human naive pluripotent stem cells also express *Lef1* and show a similar requirement for titrated CH (Takashima et al., 2014).

Rat ESCs can be exploited as an *in vitro* differentiation system, complementary to mouse and human pluripotent stem cells. Knockin reporters are extremely useful tools in this context. For example, the *Sox10* reporter generated here could be exploited for monitoring differentiation into neural crest or oligodendroglia, and for purifying desired cell populations.

ESC-mediated genome engineering has been transformative in mouse genetics and now provides similar opportunities in the rat, which in several areas of physiology and neuroscience has considerable advantages over the mouse as a model species. The *Sox10::dsRed* rat model generated here can facilitate study of neuron-oligodendrocyte interactions and remyelination. Importantly, the rat is preferred to the mouse in this context. Notably rats are used for the cerebellar caudal peduncle (CCP) ethidium bromide model of myelin regeneration (Goudarzvand et al., 2016; Woodruff and Franklin, 1999). The CCP is one of the few fully myelinated tracts in the brain and is often affected by demyelinating disease, such as multiple sclerosis (Preziosa et al., 2014). Rats are used for this lesion because the CCP is not accessible to surgery in mice. More generally, neuropharmacology, cellular distribution of neurotransmitter receptors, and neurotransmitter receptor structure are more similar between humans and rats (Hirst et al., 2003). The *Sox10* reporter rat may also be useful in investigations of white matter plasticity, taking advantage of the repertoire of behavioral assays available for rats.

In conclusion, these findings demonstrate that CRISPR/Cas9 methodology can readily be implemented in rat ESCs. Genome editing in rat ESCs constitutes a powerful system for comparative molecular genetic dissection of *in vitro* pluripotent stem cell biology. More broadly, the capacity for germline transmission provides a platform for generating advanced animal models in this important species for biomedical research.

EXPERIMENTAL PROCEDURES

Production of Rat Chimeras

Blastocyst microinjection was carried out as previously described (Blair et al., 2012) using host blastocysts from the albino Sprague-Dawley strain. Chimeras were identified by mixed coat color. All animal studies were approved by the UK Home Office and carried out in a designated facility.

Cell Culture

Rat ESCs were derived from E4.5 blastocysts from the Dark Agouti strain and maintained on γ-irradiated mouse embryo fibroblasts in aN2B27 medium supplemented with t2iL + Y, consisting of MEK inhibitor PD0325901 (1 μM), GSK3 inhibitor CHIR99021 (1 μM), human recombinant leukemia inhibitory factor (10 ng/mL, prepared in house) and Rho-associated kinase inhibitor, Y-27632 (5 μM). Cultures were maintained in a humidified incubator at 37°C in 7% CO_2 and 5% O_2. To prepare aN2B27, we used advanced DMEM/F12 (Gibco, catalog no. 12491015). Rat ESCs were routinely passaged by dissociation into single cells with TrypLE Express every 48 hr and replated at a split ratio between 1:4 and 1:6. Sufficient culture medium was added when seeding the plates that no change was required until the next passage.

Gene Targeting via CRISPR/Cas9

A newly derived rat ESC line, DAC27, was used at passage 8 for gene targeting experiments. gRNAs were designed using the CRISPR Design tool (http://crispr.mit.edu/) to target the desired region (Table S1). For *Lef1* targeting, 1 × 10^6 rat ESCs maintained in aN2B27 (t2iL + Y, 5% O_2) were transfected using Lipofectamine 2000 with 1.2 μg of expression plasmid containing gRNA, Cas9, and GFP (pSpCas9(BB)-2A-GFP (PX458), a gift from Feng Zhang; Addgene plasmid no. 48138) (Ran et al., 2013). Eight hours post transfection, cells were replated onto new feeders in fresh medium. Twenty-four hours after replating, GFP-positive cells were sorted by fluorescence-activated cell sorting onto 10 cm culture dishes at a density of 10,000 cells per dish. Fifteen milliliters of medium was added into each dish and no medium change was required thereafter. Five days after

Figure 4. Imaging and and Patch-Clamp Characterization of Sox10-dsRed-Positive Cells in Brain Slices

(A) Representation of a cortical Sox10-dsRed rat coronal brain slice. Magnification of red dashed insert shows the live detection of $dsRed^+$ oligodendrocyte lineage cells in the rat cortex. Magnification of the black dashed insert represents the live detection of $dsRed^+$ oligodendrocyte lineage cells in the rat white matter (corpus callosum).

(B–E) Schematic and imaging representation of live dsRed detection with simultaneous dye-filling with lucifer yellow (LY) of an oligodendrocyte lineage cell during whole-cell patch-clamp recordings (B). Scale bars represent 10 μm. Characteristic I-V and voltage-gated Na_v expression levels in dsRed-$Sox10^+$ (C) early oligodendrocyte progenitor cells, (D) mature oligodendrocyte progenitors, and (E) oligodendrocytes.

(F and G) Representative response of dsRed-$Sox10^+$ rat oligodendrocyte lineage cells to kainate (30 μM) (F) and the percentage of responsive and non-responsive DsRed-$Sox10^+$ rat oligodendrocyte lineage cells to kainate (G). Total number of cells used for (G), n = 17 from N = 4 separate biological replicates.

seeding, individual colonies were picked, expanded briefly, and screened using genomic PCR. For generation of *Sox10* knockin, 1×10^6 cells were transfected with 1.2 μg of gRNA plasmid, 1.2 μg of Cas9 nickase plasmid, and 1.2 μg of targeting vector. We used circular plasmids to minimize random integration. Eight hours post transfection, cells were replated onto 4 × 10 cm culture dishes and G418 selection commenced 24 hr later. Medium was replaced every 24 hr for the first 4 days and every 48 hr thereafter. To ensure robust attachment of rat ESCs during selection, a thin layer of Matrigel (BD Matrigel, 1:240 dilution in MEF medium) was applied to the MEF feeder layer 24 hr before rat ESC seeding. After 1 week of selection, individual colonies were picked, expanded briefly, and screened by genomic PCR. To confirm *Lef1* targeted clones, multiple sets of genotyping primers were used to analyze up to 2.2 kb around the gRNA recognition site, and genomic PCR products were sequenced for mutations and deletions (Table S3). To confirm *Sox10* targeted clones, genomic PCR product amplified using *Sox10 set1 (5HA)* primers was inserted into TA clones and sequenced using M13 forward and reverse primers. The region close to the *Sox10* gRNA recognition site was also sequenced using customized primer to confirm the absence of mutations.

Gene Expression Analysis by Real-Time qPCR

Total RNA was isolated using the RNeasy Kit (QIAGEN) and cDNA prepared using SuperScriptIII (Invitrogen) and 3′RACE adapter primers. Primers and probes used for real-time PCR are listed in Table S2.

Chromosome Analysis

Cells were treated for 2.5 hr with colcemide (Gibco, 1:100 dilution) 24 hr after passaging. Metaphase chromosome spreads were prepared and imaged at 63×. Chromosomes in discrete spreads were counted.

Immunofluorescence Cell Staining

Cells were fixed with 4% paraformaldehyde in PBS (pH 7.0) for 30 min at room temperature. Subsequently, cells were washed twice with PBST (0.1% Triton X-100 [Sigma] in 1× PBS) and then with blocking solution (4% donkey serum in PBST). Primary antibody solution was prepared by diluting antibody in blocking solution at the concentration listed in Table S4. Cells were incubated with the primary antibody at room temperature for 2 hr or at 4°C overnight, followed by three washes with PBS containing 0.1% Tween 20 prior to incubation with the secondary antibodies at room temperature for 1 hr. After nuclear staining with DAPI (Invitrogen), stained cells were detected by fluorescence microscopy.

Fluorescence-Activated Cell Sorting

Fluorescent E11.5 embryos were cut into small pieces before incubating in TrypLE Express enzyme for 15 min at room temperature. Digested tissue was triturated using a p1000 pipette. Enzyme was inactivated and diluted with serum containing wash buffer. Larger debris was removed with 100 μm cell strainers before re-suspension in 1 mL of PBS containing 2% BSA for sorting using a Bio-Rad S3 cell sorter.

Immunostaining of Rat Brain

Sox10::dsRed rats at 14 weeks were perfused with 4% paraformaldehyde, and coronal sections cut on a vibratome (100 mm). Fixed slices were incubated for 5 hr in 0.5% Triton X-100, 10% goat serum in PBS at 21°C, then with primary antibody at 21°C overnight, and then for 5 hr at 21°C with secondary antibody. Cryostat sections were incubated for 1 hr in 0.1% Triton X-100, 10% goat serum in PBS at 21°C, then with primary antibody at 4°C overnight, and then for 1 hr at 21°C with secondary antibody. Primary antibodies were: rabbit or mouse RFP (Abcam, 1:100), mouse NG2 (Millipore, 1:100), rabbit Olig2 (Millipore, 1:300), rabbit MBP (Sigma, 1:100). Secondary antibodies were goat anti-rabbit IgG and anti-mouse IgG (Life Technologies, 1:1,000). DAPI (Sigma) was used to label nuclei (10 min, 1 μg/mL).

Electrophysiology

Parasagittal cerebellar slices (225 μm) were prepared from P3-10 *Sox10::dsRed* rats using a vibrating blade microtome (Leica VT1200S). After dissection, the brain was placed in a cooled (~1°C) oxygenated (95% O_2/5% CO_2) Krebs solution containing: 126 mM NaCl, 24 nM $NaHCO_3$, 1 mM NaH_2PO_4, 2.5 mM KCl, 2.5 mM $CaCl_2$, 2 mM $MgCl_2$, 10 mM D-glucose (pH 7.4). Kynurenic acid was included to block glutamate receptors, which might be activated during the dissection procedure and cause cell damage. During experiments, slices were superfused with HEPES-buffered external solution containing: 144 mM NaCl, 2.5 mM KCl, 10 mM HEPES, 1 mM NaH_2PO_4, 2.5 mM $CaCl_2$, 10 mM glucose, 0.1 mM glycine (to co-activate NMDA receptors), 0.005 mM strychnine (to block glycine receptors). pH was set to 7.4 with NaOH and the solution was permanently bubbled with 100% O_2. Recording electrodes were filled with an internal solution comprising: 130 mM K-gluconate, 4 mM NaCl, 0.5 mM $CaCl_2$, 10 mM HEPES, 10 mM BAPTA, 4 mM MgATP, 0.5 mM Na_2GTP, 2 mM K-lucifer yellow, pH set to 7.3 with KOH; electrode resistance ranged from 5 to 9 MΩ. Series resistance was left uncompensated and averaged at 30 ± 1.5 MΩ. Electrode junction potential of −14 mV was compensated for. A Multiclamp 700B (Molecular Devices) was used for voltage-clamp data acquisition. Data were sampled at 50 kHz and filtered at 10 kHz using pClamp10.3 (Molecular Devices).

SUPPLEMENTAL INFORMATION

Supplemental Information includes Targeting Protocol, four figures, and four tables and can be found with this article online at http://dx.doi.org/10.1016/j.stemcr.2017.09.005.

AUTHOR CONTRIBUTIONS

Y.C. performed and interpreted experiments; S.S. performed the patch-clamp recording; and S.A. performed live imaging of dsRed+ oligodendrocyte lineage cells and prepared the figure. R.T.K. designed and supervised the electrophysiology experiments and performed immunostaining of brain sections. A.S. designed and supervised the study and wrote the paper with Y.C.

ACKNOWLEDGMENTS

We are grateful to William Mansfield and Charles-Étienne Dumeau for generation of chimeras, Sam Jameson and staff for expert

husbandry, Rosalind Drummond for help with qRT-PCR, Yasmin Paterson for help with cell culture, Peter Humphreys for imaging support, Andy Riddell for flow cytometry support, and Marko Hyvonen for recombinant LIF. This research was funded by the European Community project EURATRANS (grant no. HEALTH-F4-2010-241504), the Biotechnology and Biological Sciences Research Council of the United Kingdom (grant no. BB/H012737/1), the Swiss National Science Foundation Sinergia Program, the Louis-Jeantet Foundation, and the Isaac Newton Trust. R.T.K. was supported by a Wellcome Trust Research Career Development Fellowship (grant no. 091543/Z/10/Z) and a Lister Institute Research Prize. A.S. is a Medical Research Council Professor (grant no. G1100526/1).

Received: February 27, 2017
Revised: September 5, 2017
Accepted: September 6, 2017
Published: October 10, 2017

REFERENCES

Blair, K., Wray, J., and Smith, A. (2011). The liberation of embryonic stem cells. PLoS Genet. *7*, e1002019.

Blair, K., Leitch, H.G., Mansfield, W., Dumeau, C.E., Humphreys, P., and Smith, A.G. (2012). Culture parameters for stable expansion, genetic modification and germline transmission of rat pluripotent stem cells. Biol. Open *1*, 58–65.

Breuskin, I., Bodson, M., Thelen, N., Thiry, M., Borgs, L., Nguyen, L., Lefebvre, P.P., and Malgrange, B. (2009). Sox10 promotes the survival of cochlear progenitors during the establishment of the organ of Corti. Dev. Biol. *335*, 327–339.

Breuskin, I., Bodson, M., Thelen, N., Thiry, M., Borgs, L., Nguyen, L., Stolt, C., Wegner, M., Lefebvre, P.P., and Malgrange, B. (2010). Glial but not neuronal development in the cochleo-vestibular ganglion requires Sox10. J. Neurochem. *114*, 1827–1839.

Brinster, R.L., and Avarbock, M.R. (1994). Germline transmission of donor haplotype following spermatogonial transplantation. Proc. Natl. Acad. Sci. USA *91*, 11303–11307.

Britsch, S., Goerich, D.E., Riethmacher, D., Peirano, R.I., Rossner, M., Nave, K.-A., Birchmeier, C., and Wegner, M. (2001). The transcription factor Sox10 is a key regulator of peripheral glial development. Genes Dev. *15*, 66–78.

Buehr, M., Meek, S., Blair, K., Yang, J., Ure, J., Silva, J., McLay, R., Hall, J., Ying, Q.L., and Smith, A. (2008). Capture of authentic embryonic stem cells from rat blastocysts. Cell *135*, 1287–1298.

Capecchi, M.R. (2005). Gene targeting in mice: functional analysis of the mammalian genome for the twenty-first century. Nat. Rev. Genet. *6*, 507–512.

Chapman, K.M., Medrano, G.A., Jaichander, P., Chaudhary, J., Waits, A.E., Nobrega, M.A., Hotaling, J.M., Ober, C., and Hamra, F.K. (2015). Targeted germline modifications in rats using CRISPR/Cas9 and spermatogonial stem cells. Cell Rep. *10*, 1828–1835.

Chen, F., Pruett-Miller, S.M., Huang, Y., Gjoka, M., Duda, K., Taunton, J., Collingwood, T.N., Frodin, M., and Davis, G.D. (2011). High-frequency genome editing using ssDNA oligonucleotides with zinc-finger nucleases. Nat. Methods *8*, 753–755.

Chen, Y., Blair, K., and Smith, A. (2013). Robust self-renewal of rat embryonic stem cells requires fine-tuning of glycogen synthase kinase-3 inhibition. Stem Cell Reports *1*, 209–217.

Cho, S.W., Kim, S., Kim, J.M., and Kim, J.S. (2013). Targeted genome engineering in human cells with the Cas9 RNA-guided endonuclease. Nat. Biotechnol. *31*, 230–232.

Cong, L., Ran, F.A., Cox, D., Lin, S., Barretto, R., Habib, N., Hsu, P.D., Wu, X., Jiang, W., Marraffini, L.A., et al. (2013). Multiplex genome engineering using CRISPR/Cas systems. Science *339*, 819–823.

Esteban, M.A., Wang, T., Qin, B., Yang, J., Qin, D., Cai, J., Li, W., Weng, Z., Chen, J., Ni, S., et al. (2010). Vitamin C enhances the generation of mouse and human induced pluripotent stem cells. Cell Stem Cell *6*, 71–79.

Garneau, J.E., Dupuis, M.E., Villion, M., Romero, D.A., Barrangou, R., Boyaval, P., Fremaux, C., Horvath, P., Magadan, A.H., and Moineau, S. (2010). The CRISPR/Cas bacterial immune system cleaves bacteriophage and plasmid DNA. Nature *468*, 67–71.

Goudarzvand, M., Choopani, S., Shams, A., Javan, M., Khodaii, Z., Ghamsari, F., Naghdi, N., Piryaei, A., and Haghparast, A. (2016). Focal injection of ethidium bromide as a simple model to study cognitive deficit and its improvement. Basic Clin. Neurosci. *7*, 63–72.

Hirabayashi, M., Kato, M., Kobayashi, T., Sanbo, M., Yagi, T., Hochi, S., and Nakauchi, H. (2010a). Establishment of rat embryonic stem cell lines that can participate in germline chimerae at high efficiency. Mol. Reprod. Dev. *77*, 94.

Hirabayashi, M., Kato, M., Sanbo, M., Kobayashi, T., Hochi, S., and Nakauchi, H. (2010b). Rat transgenesis via embryonic stem cells electroporated with the Kusabira-orange gene. Mol. Reprod. Dev. *77*, 474.

Hirabayashi, M., Tamura, C., Sanbo, M., Kato-Itoh, M., Kobayashi, T., Nakauchi, H., and Hochi, S. (2013). A retrospective analysis of germline competence in rat embryonic stem cell lines. Transgenic Res. *22*, 411–416.

Hirabayashi, M., Goto, T., Tamura, C., Sanbo, M., Hara, H., and Hochi, S. (2014). Effect of leukemia inhibitory factor and forskolin on establishment of rat embryonic stem cell lines. J. Reprod. Dev. *60*, 78–82.

Hirst, W.D., Abrahamsen, B., Blaney, F.E., Calver, A.R., Aloj, L., Price, G.W., and Medhurst, A.D. (2003). Differences in the central nervous system distribution and pharmacology of the mouse 5-hydroxytryptamine-6 receptor compared with rat and human receptors investigated by radioligand binding, site-directed mutagenesis, and molecular modeling. Mol. Pharmacol. *64*, 1295–1308.

Hwang, W.Y., Fu, Y., Reyon, D., Maeder, M.L., Kaini, P., Sander, J.D., Joung, J.K., Peterson, R.T., and Yeh, J.R. (2013). Heritable and precise zebrafish genome editing using a CRISPR-Cas system. PLoS One *8*, e68708.

Iannaccone, P.M., and Jacob, H.J. (2009). Rats!. Dis. Model. Mech. *2*, 206–210.

Jacob, H.J., Lazar, J., Dwinell, M.R., Moreno, C., and Geurts, A.M. (2010). Gene targeting in the rat: advances and opportunities. Trends Genet. *26*, 510–518.

Kelsh, R.N. (2006). Sorting out Sox10 functions in neural crest development. Bioessays *28*, 788–798.

Kessaris, N., Fogarty, M., Iannarelli, P., Grist, M., Wegner, M., and Richardson, W.D. (2006). Competing waves of oligodendrocytes in the forebrain and postnatal elimination of an embryonic lineage. Nat. Neurosci. *9*, 173–179.

Kim, H., and Kim, J.S. (2014). A guide to genome engineering with programmable nucleases. Nat. Rev. Genet. *15*, 321–334.

Li, P., Tong, C., Mehrian-Shai, R., Jia, L., Wu, N., Yan, Y., Maxson, R.E., Schulze, E.N., Song, H., Hsieh, C.L., et al. (2008). Germline competent embryonic stem cells derived from rat blastocysts. Cell *135*, 1299–1310.

Li, D., Qiu, Z., Shao, Y., Chen, Y., Guan, Y., Liu, M., Li, Y., Gao, N., Wang, L., Lu, X., et al. (2013a). Heritable gene targeting in the mouse and rat using a CRISPR-Cas system. Nat. Biotechnol. *31*, 681–683.

Li, W., Teng, F., Li, T., and Zhou, Q. (2013b). Simultaneous generation and germline transmission of multiple gene mutations in rat using CRISPR-Cas systems. Nat. Biotechnol. *31*, 684–686.

Lieber, M.R. (2010). The mechanism of double-strand DNA break repair by the nonhomologous DNA end-joining pathway. Annu. Rev. Biochem. *79*, 181–211.

Ma, Y., Zhang, X., Shen, B., Lu, Y., Chen, W., Ma, J., Bai, L., Huang, X., and Zhang, L. (2014). Generating rats with conditional alleles using CRISPR/Cas9. Cell Res. *24*, 122–125.

Mali, P., Yang, L., Esvelt, K.M., Aach, J., Guell, M., DiCarlo, J.E., Norville, J.E., and Church, G.M. (2013). RNA-guided human genome engineering via Cas9. Science *339*, 823–826.

Marraffini, L.A., and Sontheimer, E.J. (2010). Self versus non-self discrimination during CRISPR RNA-directed immunity. Nature *463*, 568–571.

Meek, S., Buehr, M., Sutherland, L., Thomson, A., Mullins, J.J., Smith, A.J., and Burdon, T. (2010). Efficient gene targeting by homologous recombination in rat embryonic stem cells. PLoS One *5*, e14225.

Meek, S., Wei, J., Sutherland, L., Nilges, B., Buehr, M., Tomlinson, S.R., Thomson, A.J., and Burdon, T. (2013). Tuning of beta-catenin activity is required to stabilize self-renewal of rat embryonic stem cells. Stem Cells *31*, 2104–2115.

Men, H., Bauer, B.A., and Bryda, E.C. (2012). Germline transmission of a novel rat embryonic stem cell line derived from transgenic rats. Stem Cells Dev. *21*, 2606–2612.

Men, H., and Bryda, E.C. (2013). Derivation of a germline competent transgenic Fischer 344 embryonic stem cell line. PLoS One *8*, e56518.

Paratore, C., Eichenberger, C., Suter, U., and Sommer, L. (2002). Sox10 haploinsufficiency affects maintenance of progenitor cells in a mouse model of Hirschsprung disease. Hum. Mol. Genet. *11*, 3075–3085.

Preziosa, P., Rocca, M.A., Mesaros, S., Pagani, E., Drulovic, J., Stosic-Opincal, T., Dackovic, J., Copetti, M., Caputo, D., and Filippi, M. (2014). Relationship between damage to the cerebellar peduncles and clinical disability in multiple sclerosis. Radiology *271*, 822–830.

Rajendran, G., Dutta, D., Hong, J., Paul, A., Saha, B., Mahato, B., Ray, S., Home, P., Ganguly, A., Weiss, M.L., et al. (2013). Inhibition of protein kinase C signaling maintains rat embryonic stem cell pluripotency. J. Biol. Chem. *288*, 24351.

Ran, F.A., Hsu, P.D., Wright, J., Agarwala, V., Scott, D.A., and Zhang, F. (2013). Genome engineering using the CRISPR-Cas9 system. Nat. Protoc. *8*, 2281–2308.

Rinholm, J.E., Hamilton, N.B., Kessaris, N., Richardson, W.D., Bergersen, L.H., and Attwell, D. (2011). Regulation of oligodendrocyte development and myelination by glucose and lactate. J. Neurosci. *31*, 538–548.

Rong, Z., Zhu, S., Xu, Y., and Fu, X. (2014). Homologous recombination in human embryonic stem cells using CRISPR/Cas9 nickase and a long DNA donor template. Protein Cell *5*, 258–260.

Shao, Y., Guan, Y., Wang, L., Qiu, Z., Liu, M., Chen, Y., Wu, L., Li, Y., Ma, X., Liu, M., et al. (2014). CRISPR/Cas-mediated genome editing in the rat via direct injection of one-cell embryos. Nat. Protoc. *9*, 2493–2512.

Shen, B., Zhang, J., Wu, H., Wang, J., Ma, K., Li, Z., Zhang, X., Zhang, P., and Huang, X. (2013). Generation of gene-modified mice via Cas9/RNA-mediated gene targeting. Cell Res. *23*, 720–723.

Shibata, S., Yasuda, A., Renault-Mihara, F., Suyama, S., Katoh, H., Inoue, T., Inoue, Y.U., Nagoshi, N., Sato, M., Nakamura, M., et al. (2010). Sox10-Venus mice: a new tool for real-time labeling of neural crest lineage cells and oligodendrocytes. Mol. Brain *3*, 31.

Simon, C., Lickert, H., Gotz, M., and Dimou, L. (2012). Sox10-iCreERT2 : a mouse line to inducibly trace the neural crest and oligodendrocyte lineage. Genesis *50*, 506–515.

Stolt, C.C., Rehberg, S., Ader, M., Lommes, P., Riethmacher, D., Schachner, M., Bartsch, U., and Wegner, M. (2002). Terminal differentiation of myelin-forming oligodendrocytes depends on the transcription factor Sox10. Genes Dev. *16*, 165–170.

Storici, F., Snipe, J.R., Chan, G.K., Gordenin, D.A., and Resnick, M.A. (2006). Conservative repair of a chromosomal double-strand break by single-strand DNA through two steps of annealing. Mol. Cell. Biol. *26*, 7645–7657.

Takashima, Y., Guo, G., Loos, R., Nichols, J., Ficz, G., Krueger, F., Oxley, D., Santos, F., Clarke, J., Mansfield, W., et al. (2014). Resetting transcription factor control circuitry toward ground-state pluripotency in human. Cell *158*, 1254–1269.

Tong, C., Li, P., Wu, N.L., Yan, Y., and Ying, Q.L. (2010). Production of p53 gene knockout rats by homologous recombination in embryonic stem cells. Nature *467*, 211–213.

Verkhratsky, A., and Steinhauser, C. (2000). Ion channels in glial cells. Brain Res. Brain Res. Rev. *32*, 380–412.

Wang, H., Yang, H., Shivalila, C.S., Dawlaty, M.M., Cheng, A.W., Zhang, F., and Jaenisch, R. (2013). One-step generation of mice carrying mutations in multiple genes by CRISPR/Cas-mediated genome engineering. Cell *153*, 910–918.

Watanabe, K., Ueno, M., Kamiya, D., Nishiyama, A., Matsumura, M., Wataya, T., Takahashi, J.B., Nishikawa, S., Nishikawa, S., Muguruma, K., et al. (2007). A ROCK inhibitor permits survival of dissociated human embryonic stem cells. Nat. Biotechnol. *25*, 681–686.

Woodruff, R.H., and Franklin, R.J. (1999). Demyelination and remyelination of the caudal cerebellar peduncle of adult rats following stereotaxic injections of lysolecithin, ethidium bromide, and complement/anti-galactocerebroside: a comparative study. Glia *25*, 216–228.

Yang, H., Wang, H., Shivalila, C.S., Cheng, A.W., Shi, L., and Jaenisch, R. (2013). One-step generation of mice carrying reporter and conditional alleles by CRISPR/Cas-mediated genome engineering. Cell *154*, 1370–1379.

Ying, Q.L., Stavridis, M., Griffiths, D., Li, M., and Smith, A. (2003). Conversion of embryonic stem cells into neuroectodermal precursors in adherent monoculture. Nat. Biotechnol. *21*, 183–186.

Ying, Q.L., Wray, J., Nichols, J., Batlle-Morera, L., Doble, B., Woodgett, J., Cohen, P., and Smith, A. (2008). The ground state of embryonic stem cell self-renewal. Nature *453*, 519–523.

Yoshimi, K., Kaneko, T., Voigt, B., and Mashimo, T. (2014). Allele-specific genome editing and correction of disease-associated phenotypes in rats using the CRISPR-Cas platform. Nat. Commun. *5*, 4240.

Yoshimi, K., Kunihiro, Y., Kaneko, T., Nagahora, H., Voigt, B., and Mashimo, T. (2016). ssODN-mediated knock-in with CRISPR-Cas for large genomic regions in zygotes. Nat. Commun. *7*, 10431.

Stem Cell Reports, Volume *9*

Supplemental Information

Gene Editing in Rat Embryonic Stem Cells to Produce *In Vitro* Models and *In Vivo* Reporters

Yaoyao Chen, Sonia Spitzer, Sylvia Agathou, Ragnhildur Thora Karadottir, and Austin Smith

Supplementary Figure 1

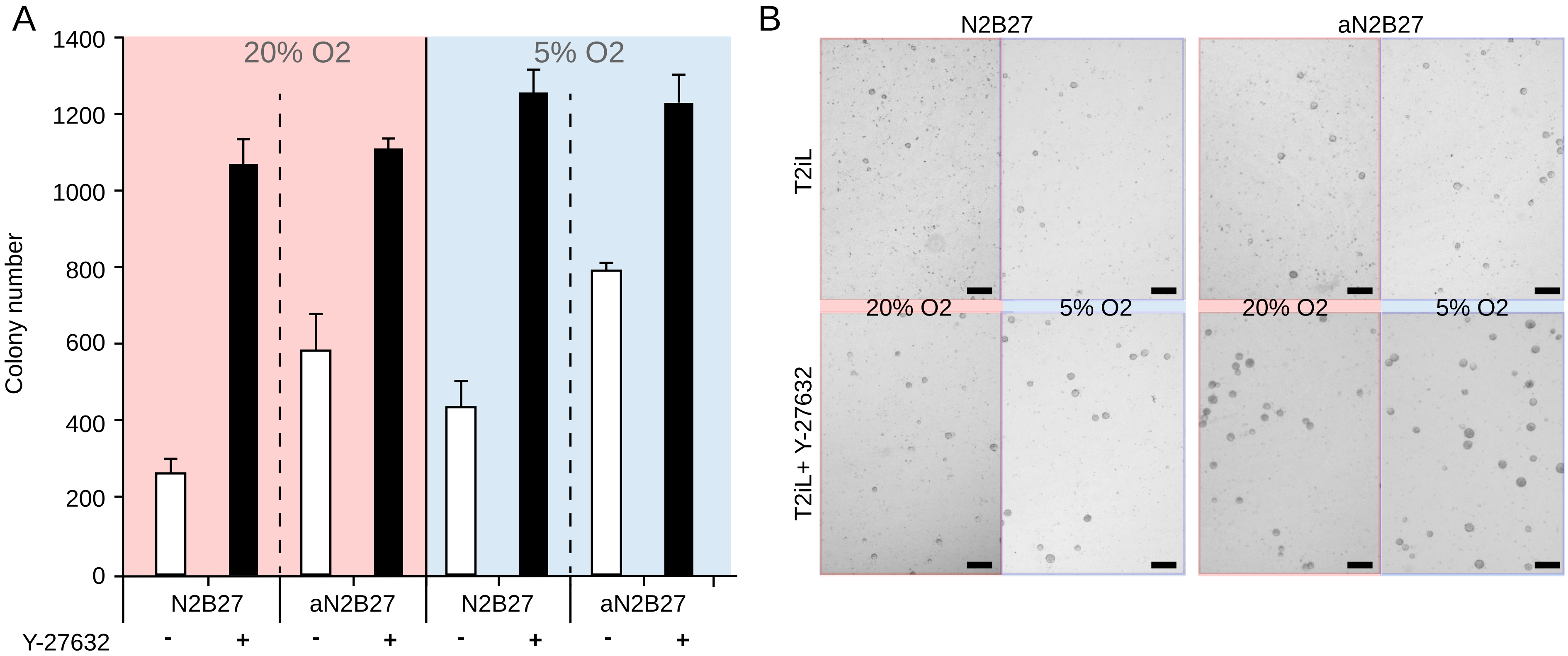

Supplementary Figure 2

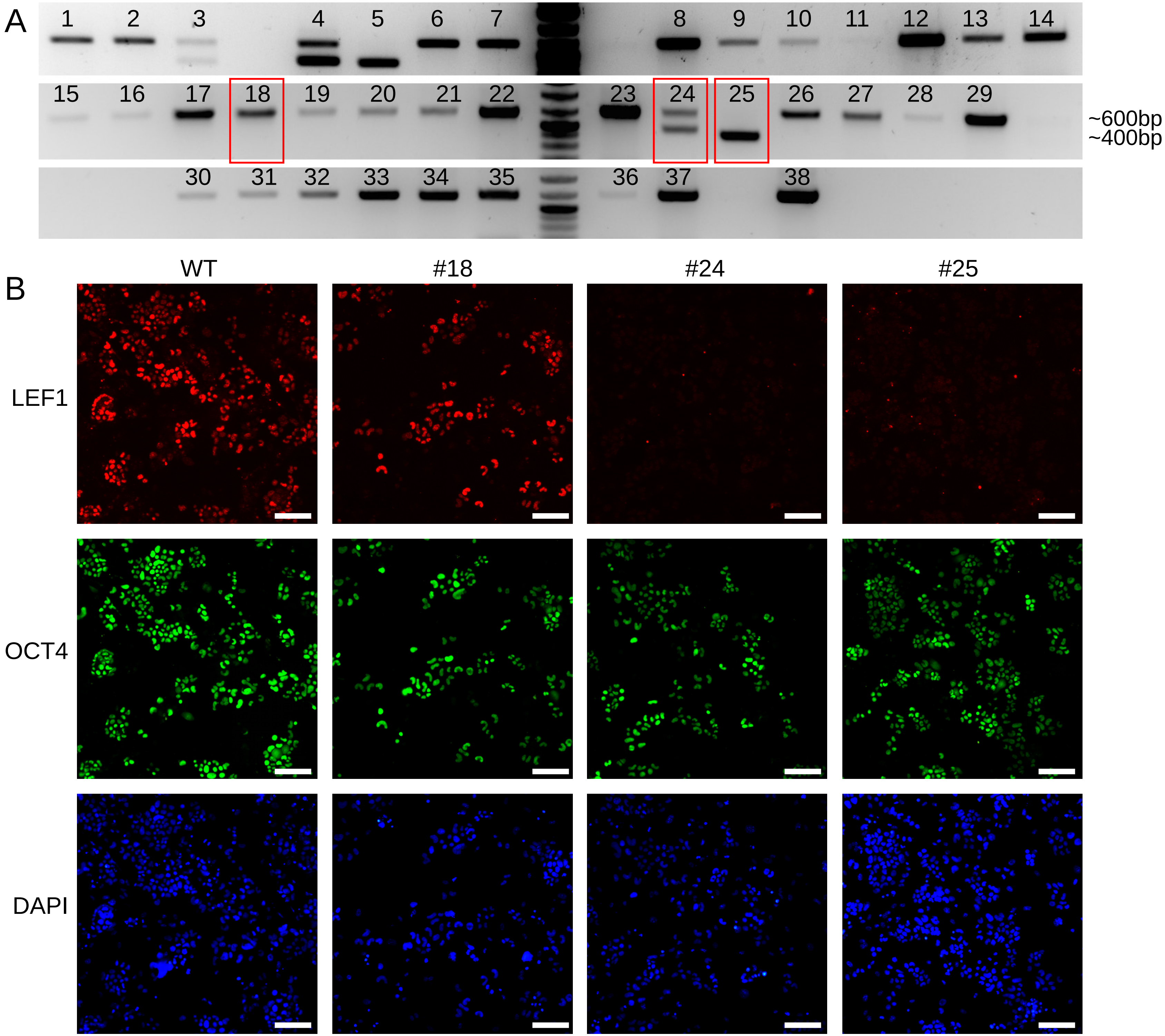

Supplementary Figure 3

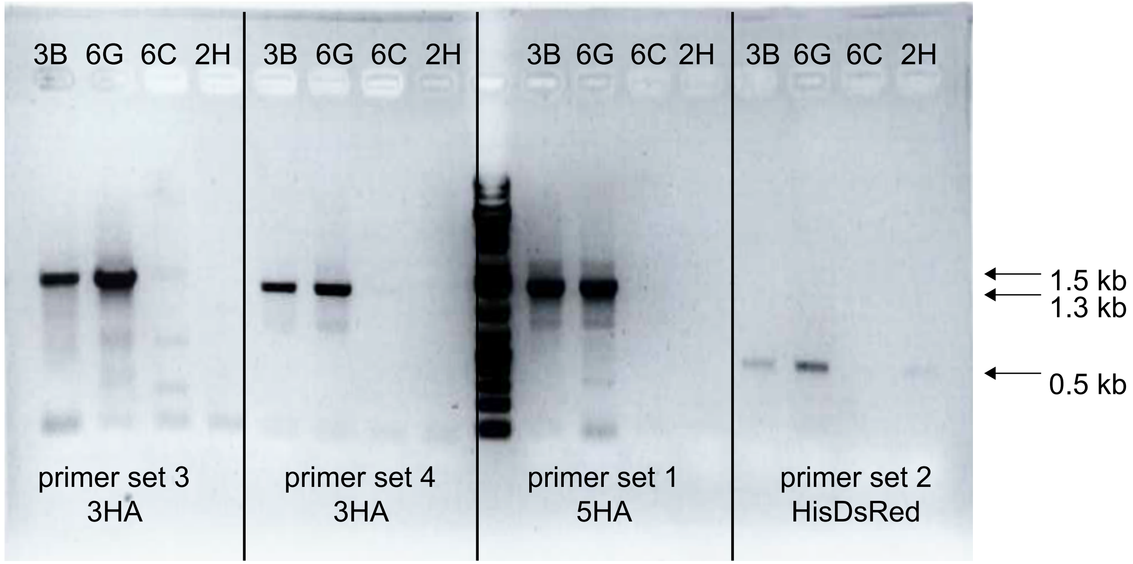

Supplementary Figure 4

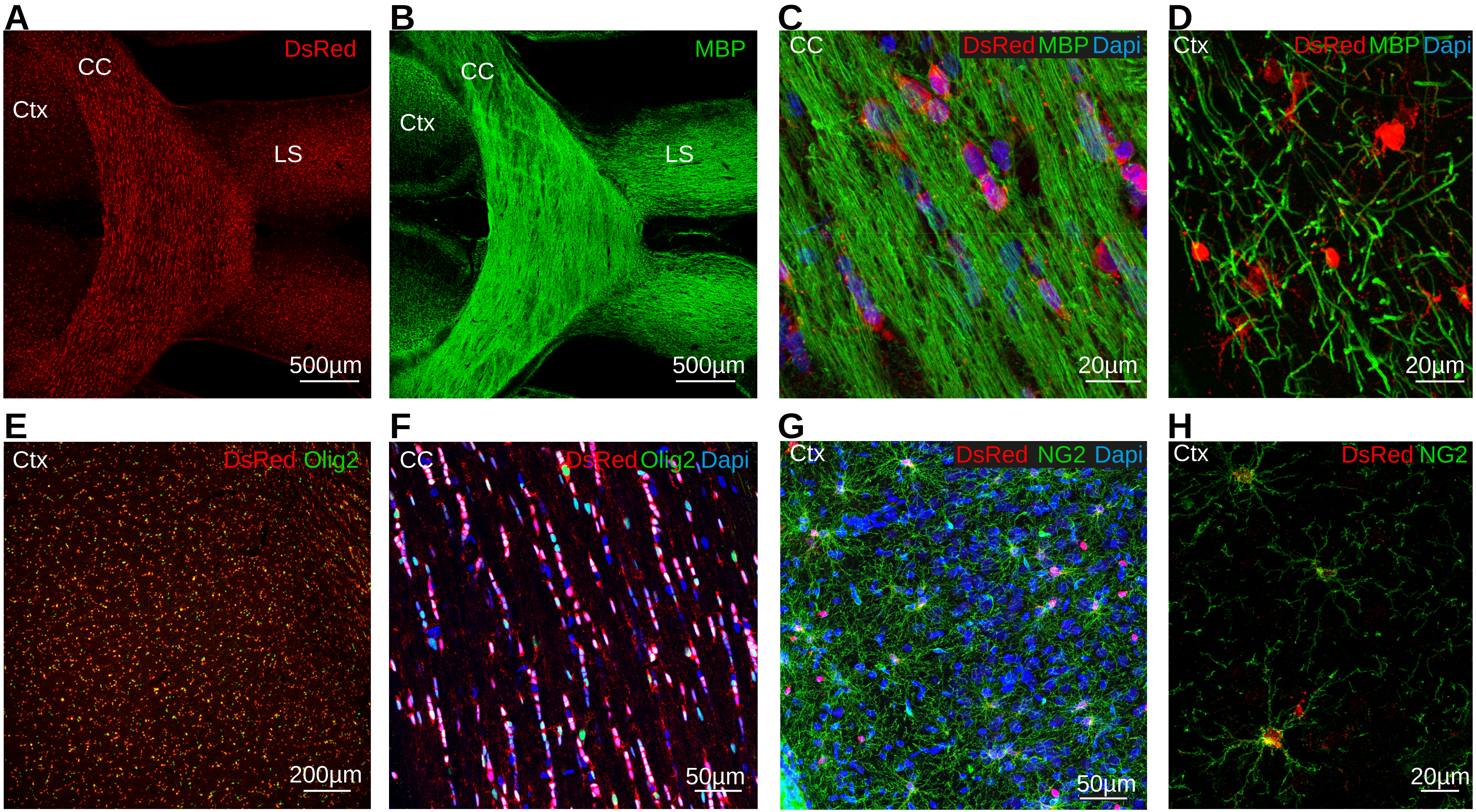

Supplementary Information

Figure S1: **Comparison of rat ES cell culture parameters**.

Related to first section of Results. (A) Colony formation in different culture conditions. For each condition, 1500 cells (per well) were seeded into 3 replicate wells in 12 well plates. The starting rat ES cell population (DAC27) was cultured in aN2B27 with Y-27632 in the presence of 5% O_2. Error bars represent SD of three biological replicates. (B) Representative images of rat ES cells in different culture conditions. Scale bars represent 200μm.

Figure S2: ***Lef1* knockout.**

Related to Figure 1. (A) Screen for *Lef1* mutation by genomic PCR. (B) Fluorescent co-staining of LEF1 and OCT4 in parental and *Lef1* mutant rES cells cultured for 24 hours on laminin coated black 24-well glass-bottom plates. Scale bars represent 100μm.

Figure S3: **Genotyping of *Sox10* targeted clones**

Related to Figure 2. Both clone 3B and 6G gave expected size bands when using indicated genotyping primer sets. Clone 6C and 2H were used as non-recombined controls.

Figure S4: **DsRed is co-expressed with oligodendrocyte lineage markers in adult rat brain**

Related to Figure 3. (A) Low magnification coronal section showing evenly distributed expression of *Sox10-DsRed* (red) in the cortex (Ctx), Corpus Callosum (CC) and subcortical areas (LS: Lateral Septal Nucleus). (B-D) Myelination appears normal in the *Sox10-DsRed* rats (Myelin Basic Protein: MBP; green, Cell nuclei: Dapi; blue, DsRed; Red), in both (C) white matter (CC) and (D) grey matter (Ctx –Layer 1) – Note that recombinant proteins are excluded from compact myelin. E. Cells expressing DsRed express the oligodendrocyte lineage marker Olig2 (Green), in both grey (E) and white matter (F). (G-H) Oligodendrocyte progenitor cells (NG2 positive cells, NG2; green) express DsRed. Scale bars are indicated on the figure.

Table S1. Guide RNA sequences

Gene	gRNA sequence
Sox10 gRNA1	CTGGCCTGCGTGGCCATAAT
Sox10 gRNA2	GACAAACACAGTCCTCTCAG
Lef1 gRNA	TCAGGAGCCCTACCACGACA

Table S2. Primers and probes for real-time PCR.

Gene	Forward primer sequence	Reverse primer sequence
Lef1	GACAAACACAGTCCTCTCAG	GCACTTTATTTGAGGTCCTCG

Gene	Company	Cat. No.
Gadph	Appliedbiosystem	4352338E
Cdx2	Appliedbiosystem	Rn00576694_m1 (Cat#:4331182)

Table S3. Primers for genomic PCR.

Gene	Forward primer sequence	Reverse primer sequence
Sox10 set1 (5HA)	gcctagcatgttctgctcgagagccc	cccctctaaggtcgggatagagt
Sox10 set2(HisDsRed)	gacaaacacagtcctctcag	gcactttatttgaggtcctcg
Sox10 set3 (3HA)	gcaaaaccaaattaagggccagc	gtgagctgagcagaaggttgga
Sox10 set4 (3HA)	taggtccctcgaagaggttcact	ggacctgaaaggagtgggtagc
Sox10 set5 (Neo)	agcgttggctacccgtgata	aaactcctcctttgccagctc
Sox10 WT	catctcacgccccagttt	tgaggtttcccaccctaccc
Lef1 set1 (671bp)	ctagaagttgggcacccagg	actgtgctgagaacccacag
Lef1 set2 (1.5kb)	gaaaactagcggggttgggt	ttcaatgtgtaggccagggg
Lef1 set3 (2 kb)	tgaactctggtggttggctt	gagacgagatgcaggtggaat
Lef1 set4 (2.2kb)	gatctctgggccagctgaag	ttcaatgtgtaggccagggg

Table S4. Primary antibodies for immunofluorescence staining.

**Antigen**	**Species**	**Dilution**	**Company**	**Cat.No.**

CDX2	Rabbit	1:200	Cell signaling	3977S
OCT4(C-10)	Mouse	1:200	Santa Cruz	Sc-5279
LEF1	Rabbit	1:500	Abcam	ab137872
SOX10	Goat	1:100	Santa Cruz	sc-17342
DsRed	Rabbit	1:100	Abcam	ab62341

Protocol for CRISPR/Cas9 facilitated gene targeting in rat embryonic stem cells

Materials

Advanced DMEM/F12 (Thermo Fisher Scientific 12634010)
B27 (Thermo Fisher Scientific 17504-044)
Beta-mercaptoethanol (Thermo Fisher Scientific 21985023)
CHIR99021 (Stemgent 04-0004)
Fetal bovine serum (Hyclone™ GE Healthcare Life Sciences SH30071.03, or equivalent)
Gelatin (Sigma-Aldrich G1890)
GMEM (Sigma-Aldrich G5154)
γ-irradiated mouse embryonic fibroblasts (iMEFs) E12.5 (prepared in house). We routinely use MEFs prepared from the multidrug resistant DR4 transgenic mice (Tucker et al., 1997)
Leukemia inhibitory factor, recombinant human (prepared in-house, but similar product is supplied by Millipore, LIF1010)
Lipofectamine® 2000 Transfection Reagent (Thermo Fisher Scientific 11668027)
L-glutamine (Sigma-Aldrich G7513)
Matrigel (Corning, 354277)
NEAA (Thermo Fisher Scientific 11140035)
Neurobasal Medium (Thermo Fisher Scientific 21103-049)
N2 supplement (Thermo Fisher Scientific 17502048)
Opti-MEM® I Reduced Serum Medium (Thermo Fisher Scientific 31985062)
Organ culture dishes (Corning 353653)
PD0325901 (Stemgent 04-0006)
Proteinase K (NEB, P8107S)
Rabbit anti-rat antiserum (Sigma-Aldrich R5256)
Rat serum (prepared in house)
Tyrode's solution, acidic (Sigma-Aldrich T1788)
TrypLE™ (Thermo Fisher Scientific 12605028)
Taq DNA polymerase (Qiagen, 201205)
Vacuum filter/storage system (Corning 431098)
Y-27632 (Dihydrochloride) (STEMCELL Technologies, 72304)

Preparation of aN2B27+t2iLY complete medium

Recipe for preparing approximately 1 litre of complete medium:

1. Thaw the N2 and B27 Supplements at room temperature (15 - 25°C) or at 2 - 8°C overnight. Ensure supplements are evenly mixed after thawing.
2. Mix 500ml of advanced DMEM/F12 and 500ml of NeuroBasal.
3. Add 5ml of N2 supplement, 10ml of B27 supplement, 10ml of 200mM L-glutamine solution, 1ml of beta-mercaptoethanol. Mix thoroughly.
4. Filter the medium using a vacuum filter/storage system (Corning™).
5. Keep the aN2B27 medium at 4°C and use within 2 months.
6. Immediately prior to use add small molecules and growth factors to reach the final concentration of 1μM PD0325901, 1μM CHIR99021, 5μM Y-27632 and 10ng/ml human recombinant LIF. The complete medium of aN2B27+t2iLY can be kept at 2 - 8°C for up to one week.

Plating inactivated mouse embryonic fibroblast (iMEF) feeder layers

1. Prepare MEF medium; GMEM with 10% FBS, NEAA, L-glutamine and 0.1mM of beta-mercaptoethanol).
2. Coat plate(s) with 0.1% gelatin and incubate at room temperature for at least 15 minutes.
3. Thaw iMEFs (commercial or prepared in house) and seed onto gelatin-coated plate(s) at a density of approximately 2.5 x 10^4 cells per cm^2. Optimal feeder layer density may vary depending on the cell preparation. Cells should cover the entire surface area of the well 48 hours after plating.
4. Incubate at 37°C overnight to allow the iMEFs to adhere.
5. iMEFs are best used within 2-7 days of plating.

Derivation of rat ES cell lines

The following protocol, modified from (Blair et al., 2012) has been used to derive ES cell lines from Dark Agouti (DA), Wistar and Fischer 344rats. Time-mated rats were purchased from Charles River or matings were set up in house. Females were euthanised on the morning of the 5th day post-coitum (E4.5).

1. Prepare a 96 well plate of iMEF at least one day before derivation and incubate at 37°C (5% O_2, 7% CO_2).
2. On the day of derivation, prepare four organ culture dishes containing aN2B27 in the central well and PBS in the outer well and pre-equilibrate in a humidified incubator at 37°C (5% O_2, 7% CO_2). Prepare and pre-equilibrate one fresh organ culture dish containing aN2B27 + 40% anti-rat serum.
3. Flush embryos from uterus at E4.5 and inspect the embryos. They should be at the mid-blastocyst stage prior to hatching.
4. Prepare a fine drawn pipette using Bunsen burner. Heat the Pasteur pipette in the middle of the thin tube until it glows orange and becomes pliable. Quickly pull the ends of the pipette away from each other to form a straight drawn pipette. Use a forceps to break the tip of the fine drawn pipette under the microscope. The size of the tip should be slightly larger than an E4.5 blastocyst.
5. Place all embryos into a pre-equilibrated organ culture dish containing aN2B27 using a fine drawn pipette. Ensure minimal carry-over of media at each transfer step.
6. Transfer less than 10 embryos at a time into a drop of acidic tyrodes solution in a sterile cell culture dish to remove the zona pellucida. Monitor dissolution of the zona under the dissecting microscope. It should take less than one minute, providing only minimal medium has been carried over.
7. Rinse the embryos in pre-equilibrated aN2B27 and transfer to the dish containing aN2B27 + 40% anti-rat serum.
8. Incubate for 1 hour.
9. Prepare and pre-equilibrate a dish of aN2B27 + 40% rat serum
10. Rinse embryos three times in pre-equilibrated aN2B27.
11. Transfer embryos to dish containing 40% rat serum.
12. Incubate for 20 minutes.
13. Inspect the embryos. The trophoblast layer should have a collapsed and largely disintegrated appearance.
14. Transfer embryos to individual droplets of aN2B27 in a sterile cell culture dish. Remove lysed trophectoderm cells mechanically by trituration using a fine pipette. Lysed trophectoderm can be reserved for genotyping to determine gender.
15. Inspect the immunosurgically isolated ICMs and deposit individually into single wells of a 96 well plate on iMEF in 100 μl of aN2B27 + t2iLY. Add 100 I.U./mL penicillin and 100 (μg/mL) streptomycin. Allow ICM explants to expand for 5-6 days at 37°C (5% O_2, 7% CO_2). Add fresh 100 μl of aN2B27 + t2iLY (without antibiotics) to each well at day 3.

16. At day 5-6, wells containing outgrowths are individually passaged 1:1 into new wells of a MEF coated 96 well plate. For passaging, aspirate the medium and add 50 μl/well of TrypLE™ Express into each well and incubate the plate at 37°C for 3 minutes. Add 150 μl /well of wash buffer (DMEM/F12 + 2% serum) and pipette up and down to stop trypsinisation and dissociate the outgrowth into single cells or doublets. Transfer cell suspension into a 96-well V-bottom plate and spin down using a plate centrifuge. Resuspend each cell pellet in 200 μl of culture medium (aN2B27+t2iLY) and seed into the new plate.
17. Colonies with domed morphology should become visible within 3 days after passaging and can subsequently be passaged every 2-3 days at ratio of 1:2 to 1:4. Gradually expand lines to progressively bigger wells. At early passages cells grow better plated into small wells.
18. Expanding cultures may be considered established ES cell lines from passage 4 onwards. Note that each line is derived from a single ICM outgrowth.
19. Freeze using medium containing 10% DMSO, 10% serum and 80% rat ES cell culture medium.

Passaging established rat ES cells

Rat ES cells are normally passaged every 48 hours with no medium change is required in between. Always passage before confluence and never allow cultures to over-grow, as cell viability will drop significantly and karyotypic changes may ensue. When assessing confluency, always check the edges of the well since these areas usually have higher cell density. The colonies are ready to be passaged when they reach 50 - 70 μm in diameter. A healthy colony has a smooth round surface. If colonies develop a roughened surface (see arrowed colonies in Figure) cells will not recover well after passaging. Therefore, if raspberry-like colonies appear in the culture, passage immediately. 12-well plates are recommended for routine rat ES cell maintenance because minimal circulation of medium reduces colony detachment.

Figure. Healthy (left) and unhealthy (right) rat ES cell cultures (scale bar: 100μm)

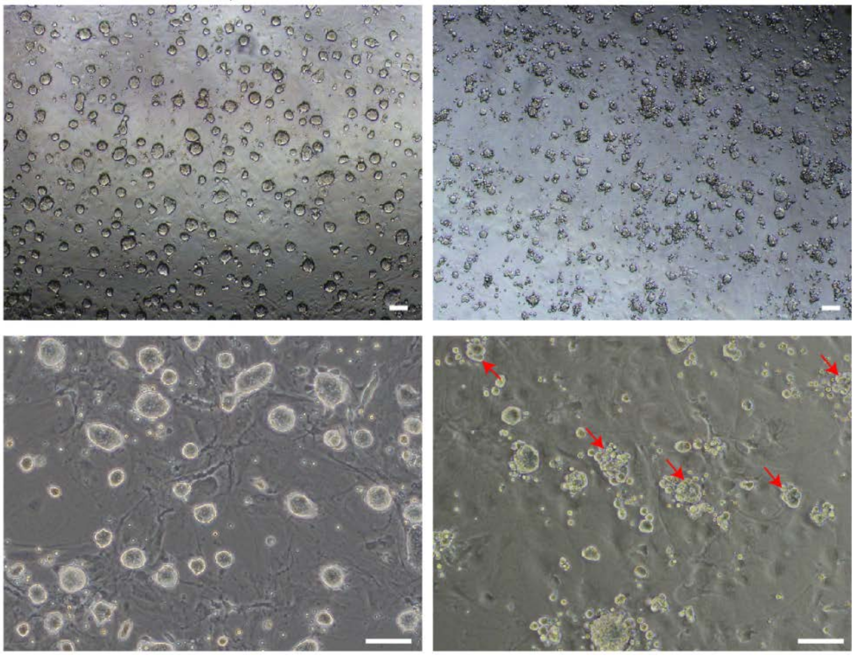

12-well plates are recommended for routine rat ES cell maintenance because minimal circulation of medium reduces colony detachment.

1. Prepare iMEF-coated plates at least one day prior to plating ES cells.

2. Warm aN2B27+t2iLY and wash buffer to room temperature before use.
3. Inspect rat ES cell culture before passaging. If significant detachment of colonies from the feeder layer is observed, collect all the culture medium from the well and spin at 300 x *g* for 3 minutes to recover those detached colonies. Resuspend them in TrypLE.
4. Otherwise, aspirate culture medium from wells containing rat ES cells. Try to remove as much residual medium as carefully as possible. It is not recommended to wash the culture with PBS since rat ES cells are loosely attached and can be lost during the wash step.
5. Add TrypLE and incubate at 37°C for 3 minutes. Recovered floating colonies can be added back to the wells they were harvested from. Allow them to be incubated at 37°C for an extra 1.5 minutes. Pipette up and down to dissociate the colonies. Try to avoid bubbles during pipetting.
6. Transfer to a 15mL conical tube. Add 4 volumes of wash buffer to neutralise TrypLE and centrifuge at 300 x *g* for 3 minutes.
7. Aspirate the wash buffer. Resuspend the cell pellet in 1mL of aN2B27+t2iLY medium.
8. Aspirate MEF medium from iMEF-coated plate. Try to remove as much MEF medium as possible.
9. Dispense rat ES cell suspension into the wells according to desired passage ratio. Top up aN2B27+t2iLY medium to 0.5mL per cm^2 (approximately 2mL per well of a 12 well plate. A healthy rat ES cell culture will typically be passaged every 2 days at a split ratio of 1:4.
10. Distribute rat ES cells evenly across the well by moving the plate in several short, back-and-forth and side-to-side motions. Incubate at 37°C (5% O2, 7% CO2).
11. Visually assess cultures daily to monitor growth until the next passage. Avoid shaking the plate as colonies may detach.

Lipofection and selection
Rat ES cells can be transfected using different methods. Following is a protocol using Lipofectamine® 2000. Note that less DNA is used for CRISPR experiments than standard transgene transfection in order to minimise undesired random integration. Plan your CRISPR constructs; design sgRNAs using tools such as http://crispr.mit.edu:8079/. Choose an expression vector to clone sgRNA-encoding plasmids. For example, we used U6-BsaI-sgRNA backbone (kindly provided by S. Gerety, Sanger Institute, Cambridge, UK). For Cas9-encoding plasmids, we chose hCas9_D10A Cas9 (a gift from George Church (Addgene plasmid # 41816)), which expresses Cas9 nickase.

1. Prepare a 12-well plate of rat ES cells (approximately 1 x 10^6 cells in total) for transfection.
2. Plate a full 6-well plate of iMEFs and four 10-cm dishes of DR4 (resistant to neomycin, hygromycin, puromycin and 6-thioguanine) iMEFs at least 1 day prior to transfection.
3. Prepare Matrigel coating on iMEF dishes. Matrigel provides better attachment of rat ES cells during selection. Add 100 μL of Corning Matrigel hESC-Qualified Matrix to 24mL of cold MEF medium and mix well. Aspirate the MEF medium from the dishes and add 6 mL of Matrigel containing medium to each dish. Incubate at 37°C for at least 8 hours before use.
4. For transfection prepare six aliquots of 200 μL Opti-MEM® I Reduced Serum Medium in Eppendorf tubes.
5. Add 12 μL of Lipofectamine® 2000 Reagent into each tube. Tap tube to mix well.
6. Prepare a mixture of 1.2 μg of sgRNA plasmid, 1.2μg of Cas9 plasmid, 1.2μg of targeting vector in 1.3 mL of Opti-MEM® I Reduced Serum Medium in a new Eppendorf tube. Pipette to mix well.

7. Dispense 200 µL of plasmid mixture into each tube of Lipofectamine® 2000 Reagent mixture. Tap tube to mix well. Incubate at room temperature for 20 minutes.
8. Dissociate rat ES cells using TrypLE as for routine passaging and transfer cell suspension 1:1 onto a new 6-well plate of iMEF.
9. Immediately add 400 µL of DNA-lipid complex dropwise to each well of the plate. Move the plate in several short, back-and-forth and side-to-side motions then return to incubator (5% O2, 7% CO2). Incubate for 8-10 hours before passaging. Please note that incubation in lipofection media for longer than 10 hours will result in decreased cell viability.
10. Passage transfected rat ES cells onto four Matrigel-coated 10cm DR4 iMEF dishes.
11. Start selection 24 hours after passaging using lowest drug concentration that completely eliminates wildtype cells.
12. Perform daily medium change for the first 4 days and subsequently change the medium every other day until the colonies are large enough for picking.

Colony picking and expansion

It usually takes 7-8 days from the start of the selection for colonies to reach 100 to 150µm in diameter, which is optimal for picking. Colonies smaller than this may be difficult to pick and may have few viable cells after picking. Larger colonies may accumulate dead cells in the middle of the colony and have higher level of spontaneous differentiation after passaging. Please note that colonies may reach size for picking on different days.

1. Plate down DR4 iMEFs on several flat-bottom 96 well plates at least 48 hours before colony picking.
2. Ideally use a fine drawn glass pipette for picking colonies. Otherwise, use a P200 pipette.
3. Prepare several U or V-bottom 96 well plates with 30 µL/well of TrypLE.
4. Pick colonies under a microscope and deposit individually into single wells of prepared 96 well plates with TrypLE. Try to pick as many colonies as possible.
5. Dispense 200 µL of wash buffer into each well and use a multi-channel pipette to pipette up and down to dissociate the colonies.
6. Spin down at 1200rpm for 3 minutes using a plate centrifuge.
7. Resuspend each cell pellet in 200 µl of complete medium supplemented with selection drug (e.g.neomycin at 300 µg/mL) and antibiotics and transfer onto DR4 iMEFs coated 96 well plates.
8. Incubate for 2-3 days. Inspect each well on day 2 and day 3. Note that different wells may require passage on different days depending on cell number and colony size.
9. Passage each well when ready into two wells on separate DR4 iMEF coated 96-well plates. Maintain selection but withdraw antibiotics. The duplicate 96-well plate is used for genotyping.
10. Once correct genotype is confirmed, gradually expand the clones from 96 well plates to progressively larger wells.

Primary genotyping

1. When the plate for genotyping becomes confluent, aspirate medium from the wells. Add 50µL of ES cell lysis buffer (100mM pH 8.5 Tris-Cl, 5mM EDTA, 0.2% SDS, 200mM NaCl, 100µg/ml Proteinase K) into each well and incubate at 55°C for at least 3 hours.
2. Add 5µL of 3M sodium acetate (pH5.2) and 100µL of ethanol into each well. Let genomic DNA precipitate at room temperature for 1-2 hours. Do not mix or shake the plate. Precipitated genomic DNA will stick to the wall of the wells. Invert the plate to decant the solution. Gently wash the well with 70% ethanol. Invert the plate to decant the ethanol and allow the plate to air dry.

3. Dissolve genomic DNA in the wells in 100µL of TE buffer. Proceed to genomic PCR using Qiagen Taq polymerase. Avoid using high efficiency DNA polymerases for this purpose as they are likely to amplify products from non-specific binding of primers to the genome.
4. When designing genotyping primer pairs for homologous recombination, one primer must be specific to the introduced sequence while the other primer corresponds to genomic sequence flanking the homology arm. When designing genotyping primer pairs for non-homologous end joining (NHEJ), the two primers of each primer pair should flank the genomic region of the CRISPR/Cas recognition sequence. It may be necessary to design primers at varying distances from the target site to define deletions resulting from NHEJ.
5. Identify correctly targeted clones by agarose gel electrophoresis. Confirm by sequencing the PCR product.

References

Blair, K., Leitch, H.G., Mansfield, W., Dumeau, C.E., Humphreys, P., and Smith, A.G. (2012). Culture parameters for stable expansion, genetic modification and germline transmission of rat pluripotent stem cells. Biology Open *1*, 58-65.

Tucker, K.L., Wang, Y., Dausman, J., and Jaenisch, R. (1997). A transgenic mouse strain expressing four drug-selectable marker genes. Nucleic Acids Research *25*, 3745-3746.
